# A systematic meta-review of interventions to prevent and manage delirium in the Intensive Care Unit: Part 1 – Pharmacological interventions

**DOI:** 10.1186/s13054-025-05615-0

**Published:** 2025-12-30

**Authors:** Katherine L Jones, Burak Kundakci, Andrew Booth, Louise Falzon, Ben Gibbison, Maria Pufulete

**Affiliations:** 1 School of Medicine and Population Health, Sheffield Centre for Health and Related Research (SCHARR), University of Sheffield, Sheffield, UK; 2 The Centre for Musculoskeletal Research, University of Manchester, Manchester, UK; 3 Bristol Medical School, University of Bristol, Bristol, UK; 4 The Department of Cardiac Anaesthesia and Intensive Care, University Hospitals Bristol and Weston NHS Foundation Trust, Bristol, UK

**Keywords:** Delirium, Adult intensive and critical care, Review

## Abstract

**Background:**

Intensive Care Unit (ICU) delirium is a multifactorial syndrome associated with prolonged hospitalization, increased morbidity and mortality, cognitive decline, and higher healthcare costs. Many randomised controlled trials of interventions to prevent or manage ICU delirium have been combined in systematic reviews. We aimed to collate and map the meta-analysed evidence for pharmacological interventions.

**Methods:**

Eligible reviews included RCTs of any pharmacological intervention designed to prevent or manage critically ill adults with, or at risk of, ICU delirium. We searched 8 databases from inception to 26 September 2023. Two reviewers independently screened search results and confirmed eligibility of full texts. We then mapped the effects for pharmacological interventions (single or combined drugs and sedation strategies) along with the certainty of the evidence for outcomes in the Del-CorS core outcome set, ICU and hospital length of stay.

**Results:**

Of 3,381 studies, we identified 56 relevant systematic reviews reporting our outcomes (17 included in mapping). Thirteen reviews with GRADE assessments were mapped for delirium outcomes (occurrence, duration or severity), six for ICU or hospital mortality, and 15 for ICU or hospital length of stay. The α2-adrenoceptor agonist drug class (primarily dexmedetomidine) had the largest evidence base and was probably favourable over placebo for preventing or reducing delirium occurrence (moderate to high-certainty evidence; 2 systematic reviews). The α2-adrenoceptor agonist drug class (primarily dexmedetomidine) was also probably favourable over placebo for reducing ICU and hospital length of stay (moderate-certainty evidence; one systematic review). The evidence was more variable for other pharmacological comparisons. For ICU mortality, there may have been little or no difference between dexmedetomidine and a non-dexmedetomidine comparator (low certainty), while the evidence was very uncertain on hospital mortality (one systematic review). No meta-analyses reported outcomes for cognition or emotional distress. Co-interventions, in particular non-pharmacological interventions, were often incompletely reported.

**Conclusions:**

Mapped evidence suggests the α2-adrenoceptor agonist drug class (primarily dexmedetomidine), is most likely to be effective at managing delirium in the ICU. However, underlying conditions indicating or precluding intervention, and the impact of loss to follow-up, remain unclear. We found a lack of synthesised evidence for important core outcomes of cognition and emotional distress, and for deprescribing sedatives/analgesia as part of optimising sedation strategies.

**Supplementary Information:**

The online version contains supplementary material available at 10.1186/s13054-025-05615-0.

## Introduction

Delirium is an acute, fluctuating, and reversible syndrome resulting in impaired cognition and/or consciousness [1]. People with delirium have trouble maintaining attention and may have visual and auditory hallucinations [2]. While prevalence estimates vary, up to 7 in 10 patients admitted to intensive care units (ICUs) are anticipated to develop delirium, with estimates higher among mechanically ventilated patients [2, 3]. Patient risk factors for delirium include the severity of the acute illness, older age, and other comorbidities (cardiovascular risk factors, plus pre-existing impaired cognition, withdrawal from alcohol and nicotine) [4]. Risk factors relating to the management of critical illness include drugs (and dose) specific to the ICU e.g. sedatives and analgesia. The incidence of ICU delirium is expected to increase further with increasing admissions of older people living with multiple long-term conditions.

ICU delirium is associated with a higher risk of mortality, morbidity, and prolonged need for mechanical ventilation, extending ICU and hospital lengths of stay and increasing healthcare costs [2, 5–10]. There are also costs associated with longer-term sequelae, which may include dementia, other cognitive or functional decline, and increased need for long-term care [11, 12]. Multiple pharmacological and non-pharmacological interventions have been used to prevent and manage delirium in the ICU setting. Randomised controlled trials of interventions have been combined in multiple systematic reviews, meta-analyses, and network meta-analyses. However, there is a lack of overarching mapping of evidence syntheses and assessment of comprehensiveness to support future research and practice. We aimed to scope and map out the meta-analysed evidence for interventions used for prevention and management of ICU delirium. This review (Part 1) specifically addresses pharmacological interventions. Part 2 addresses non-pharmacological and multicomponent interventions. Together, these reviews are anticipated to inform future critical care research and delivery of complex interventions in the ICU setting.

## Methods

The full protocol for this meta-review was published [13] and registered previously with PROSPERO (CRD42023473260). The evidence for pharmacological and non-pharmacological studies was conceptualised as a single meta review. However, given the volume of the evidence identified, we chose to split the review into two parts: Part 1, pharmacological interventions (including sedation strategies) and Part 2, non-pharmacological interventions (including care bundles). We applied guidance from Cochrane overviews [14], the Preferred Reporting Items for Overviews of Reviews (PRIOR [15] and PRISMA Extension for Scoping Reviews (PRISMA-ScR [16] to support standardised evidence synthesis methods in the conduct and the reporting of the meta-review. We also sought input on aspects of the methodology from a Patient and Public Involvement group of people with lived experience of ICU delirium.

### Literature search

Our search included any type of pharmacological or non-pharmacological intervention and comprised a combination of subject headings and text words to represent the concepts of delirium AND intensive care AND systematic reviews. The search was performed in MEDLINE (Ovid), Embase (Elsevier), Cochrane Database of Systematic Reviews, Scopus, Cumulative Index to Nursing and Allied Health Literature (CINAHL), PsycINFO and Web of Science (from inception to 26 September 2023), as well as in initial scoping search of Epistemonikos (from inception to 19 July 2023), with manual deduplication of records.

Although we pre-specified in our inclusion criteria that we would only include studies from the year 2000 onwards, most reviews we identified were published after this date, with only a minority of included trials published before 2000. Searches were unrestricted by language but limited to English at study selection. See Additional file 1 for full details.

### Study selection and data extraction

Two of three reviewers (KLJ, BK, and AB) independently screened search results. Dual screening was completed for a subset of at least 20% of records at title/abstract and full text. One reviewer (KLJ) confirmed the eligibility of included studies based on review-level reporting, with discussion among the review team and agreement of exclusion reasons. Standardised data extraction fields included demographics, review methods, and effect estimates (Additional file 2a). One reviewer (KLJ) extracted data, and a second member of the author team (BG) crosschecked a random 10% of included reviews for data entry as well as all mapped outcomes against the extracted results, with any queries resolved by discussion. The main consideration requiring consensus related to how interventions were classified.

### Eligibility

The eligibility criteria for our mapping meta-review (embracing aspects of scoping, overview and mapping methodology) [17] are summarised in Table 1. We included sedation strategies as pharmacological interventions not multicomponent care bundles.

**Table 1:** Meta-review eligibility criteria

Criteria	Types of review	Participants and setting	Interventions
Inclusion	o Systematic review* of RCTs or mixed study designs including RCTs where separate analyses reported	o Critically ill adults (aged ≥ 18 years). Mixed patient group or single subgroup (e.g. sepsis, mechanically ventilated) o Mixed or single critical care (HDU/ICU setting e.g. burn, cardiac, medical, surgical, trauma) o Mixed critical and non-critical settings only if ≥ 80% of included studies conducted in ICU	o Any drug or combination of drugs used to prevent, treat or manage ICU delirium o Any sedation strategy used to prevent, treat or manage ICU delirium (e.g. awakening trials, deprescribing or avoidance of benzodiazepines) o Any comparator
Exclusion	o Not in English o Protocol only o Overview of reviews (references checked only) o Review with no systematic search strategy/planned synthesis	o Alcohol withdrawal o POD if not ICU o Intermediate care units (e.g. coronary care units)	o Non-pharmacological intervention o Care bundle (excluding any sedation strategy, classified here as pharmacological intervention)

RCT: randomised controlled trial; ICU: Intensive care unit; HDU: High dependency unit; POD: post-operative delirium.*Systematic reviews included literature reviews with a systematic search strategy and planned synthesis reported

### Outcome measures

We used the Del-COrS core-outcome set [18] for research evaluating interventions to prevent or treat delirium in critically ill adults, as well as additional outcomes considered important by the research team and patient/carer partners. We assessed outcomes separately for interventions designed to i) prevent, and/or ii) manage delirium (see Table 2).

**Table 2:** Primary and secondary outcome measures for the prevention, treatment or management of ICU delirium

Type of intervention	Primary outcomes	Secondary outcomes
Prevention	Delirium occurrence (including incidence and prevalence)	• Time to delirium resolution/Duration of delirium • Delirium severity • Mortality • Cognition including memory • Emotional distress (including anxiety, depression, acute stress, or post-traumatic stress disorder) • Health-related quality of life • ICU length of stay* • Hospital length of stay* • Adverse events*
Treatment or Management	Time to delirium resolution/Duration of delirium/Delirium recurrence	As above except for primary outcomes

*Additional outcomes not in Del-COrS core-outcome set

### Classifying review interventions

Reviews were classified as prevention, treatment or management, or unclear. The criteria for classification are shown in the data extraction template (Additional file 2a). Classification was based on review-level reporting and included study descriptions; management reviews (including treatment) required delirium diagnosis in selection criteria, and prevention reviews did not report selection criteria for patients with ICU delirium.

### Interpreting review comprehensiveness

Quantitative evidence was reported narratively. We applied guidance on the conduct and reporting of overviews [14], avoiding statistical comparison of effect estimates and subsequent conflation of comparisons from reviews with different eligibility criteria and different approaches to meta-analysis. We considered GRADE assessment [19] and the availability of network meta-analyses as key indicators of review comprehensiveness for outcome mapping of the meta-analysed evidence. Other considerations for review comprehensiveness during data extraction are described in the supplementary material (Additional file 2a).

### Mapping the review evidence

A mapping strategy for core outcomes from reviews was developed post-protocol to capture the direction of effect for direct comparisons that had pooled, statistical analyses and GRADE assessment. For a large and heterogenous evidence base, this allowed an overview of the level of review agreement and certainty across core outcomes and pharmacological interventions, reducing dependency on consistent review reporting for included studies (i.e. addressing aspects of review methods rather than included study overlap and volume as an indicator of comprehensiveness). If a review reported results for both pairwise and network meta-analysis, this was recorded with the latter mapped. Cardiac surgical ICU results were mapped only in the absence of ICU results, and neurological ICU results would have been mapped similarly if there had been usable results. For transparency, the maps were supported by supplementary reporting of the effect estimates with 95% confidence or credible intervals. Adverse events were broadly charted by type.

### Assessing the quality of the review evidence

Included study or outcome risk of bias and GRADE assessments were collected to inform interpretation and mapping. No further assessment was performed for the meta-review. Reasons for downgrading the mapped evidence certainty were reported narratively.

## Results

### Study selection

**Fig. 1:**
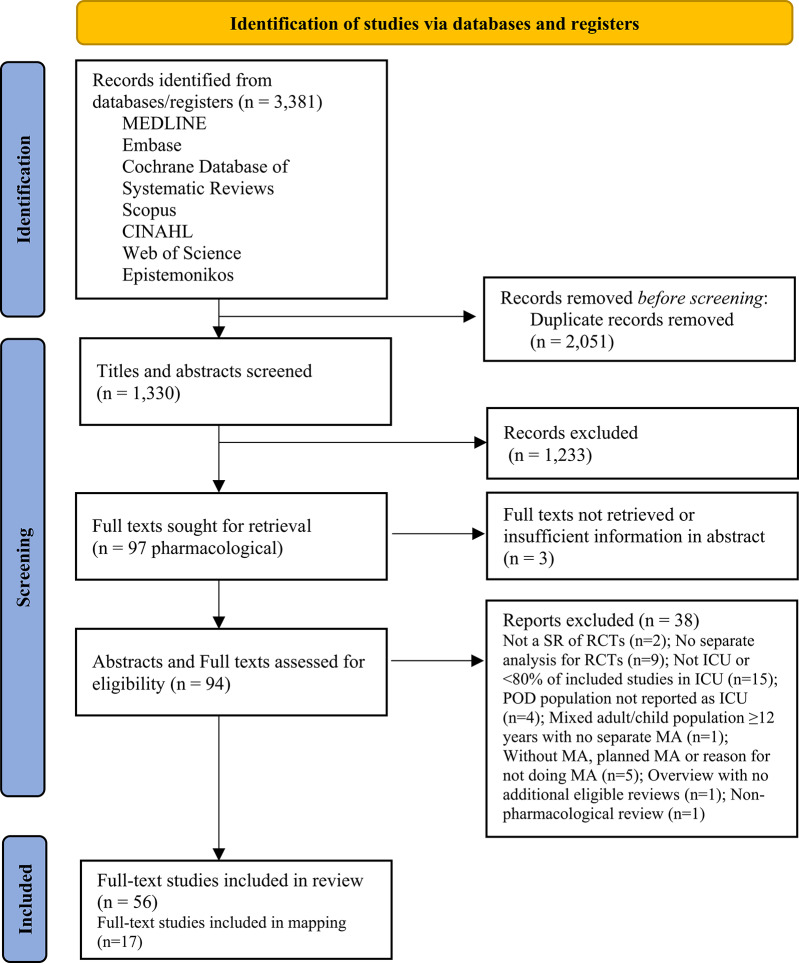
PRISMA flow diagram

Our search results are summarised in a PRISMA flow diagram (Fig. 1). See also PRIOR [15] and PRISMA-ScR checklists [16] in the Supplementary Information: Additional file 8 and 9. Electronic database searches identified 3381 citations. After removing duplicates there were 1330 unique records. We initially excluded 1233 studies at title/abstract and assessed 97 abstracts/full texts that addressed pharmacological interventions; 3 of these were subsequently excluded because full texts could not be retrieved or there was insufficient information in the abstract. During data extraction, 41 further reports were excluded with reasons given in accordance with meta-review eligibility criteria (Additional file 2b). There were 56 included full texts. These reviews are summarised in Table 3 and individual review characteristics reported in Additional files 3a and b.

**Table 3:** Summary of included reviews

Review characteristics	No. of reviews, *n* (%)
***Total number of included reviews***	56 (100)
Reviews of RCTs only	40 (71)
Reviews of RCTs and other study designs	16 (29)
***Review populations***	
Critical illness (with/without mechanical ventilation)	34 (61)
Mechanical ventilation	17 (30)
Other*	5 (9)
***Methodological design***	
Network meta-analysis	5 (9)
Pairwise meta-analysis	47 (84)
Planned meta-analysis but not done/Reason given for not doing meta-analysis	4 (7)
***Included RCTs published since 2000***	
100%	50 (89)
75–99%	6 (11)
< 75%	0 (0)

RCT: randomised controlled trial; ICU: Intensive care unit; HDU: High dependency unit.*Post-surgery with/without mechanical ventilation (3) or trauma patients (1) (ICU/HDU), or mechanically ventilated patients in a review of hospitalised patients (1)

All reviews were published after 2000 and most included RCTs only (71%). Six reviews included a small proportion (≤ 25%) of RCTs published before 2000 [20–25]. There was significant primary study overlap but discrepant reporting between reviews for the same studies (e.g. for ICU setting) precluded meaningful analysis.

### Review populations

Thirty-four of 56 reviews included a broad population of critically ill adults. Seventeen reviews focused on mechanically ventilated patients only and five reviews specified other subpopulation criteria. All but one review [26], which included a single RCT of healthy adults in a simulated ICU environment, included studies conducted in ICU/HDU settings. Broadly, the review population consisted of middle-aged to older adults except for the review of a trauma population, who were slightly younger to middle-aged patients [10]. Typically, reviews reported a higher proportion of men than women among other demographics reported (Additional file 3a).

### Delirium assessment

Across all included reviews, we identified the use of different delirium or delirium-related detection/diagnostic tools—most commonly the Confusion Assessment Method for the ICU (CAM-ICU) [6, 7, 10, 21–23, 26–55]. Other versions/tools included the Confusion Assessment Method (CAM) [33, 34, 48, 52], Intensive Care Delirium Screening Checklist (ICDSC) [6, 7, 22, 23, 27–29, 35, 37, 39, 44, 45, 50, 53, 55], Diagnostic and Statistical Manual of Mental Disorders (DSM) criteria [26, 28, 35, 37, 40, 42, 45–47], 4-point scale for delirium [38], Delirium Detection Scores [22], Cognitive Test for Delirium [22], Neelon and Champagne Confusion Scale [22], and the Delirium Rating Scale [26, 46]. Over one third of reviews did not report a delirium assessment method.

### Classifying review interventions

When we attempted to classify interventions as either prevention or treatment/management we found most reviews did not provide enough information or reported this inconsistently. One review investigated both separately [34]. We cautiously identified eight reviews that referred to intervention as prophylactic or preventative of delirium [26, 27, 34, 35, 44, 46, 53, 54], four reviews of treatment or management [20, 34, 39, 40] and the rest (n = 45; 80%) were interpreted as either/or, based on review-level reporting in accordance with our classification criteria (see Methods). It was unclear if ‘prevention’ was considered primary, secondary or tertiary. Across all included reviews, the comparator group varied between no intervention, placebo, usual care, and other intervention. Reviews also reported inconsistent usual care between trial arms, cross-over and pre-randomisation drug treatment as trial design limitations [24, 31, 37, 46, 56].

### Reporting of meta-review primary outcomes

Review outcomes are described in Additional file 3b. Delirium occurrence (incidence and prevalence) was the most reported outcome although this was inconsistently defined and incompletely reported for timing. Several reviews reported composite delirium outcomes, primarily of delirium-free and coma-free days [6, 7, 35, 41, 44, 47, 55].

### Single and multiple interventions

Commonly investigated drug classes included α2-adrenoceptor agonists (e.g. dexmedetomidine), typical and atypical antipsychotics, and melatonergics. No included review investigated the effect of deprescribing a specific drug as the intervention. However, the avoidance of benzodiazepines was explored through comparison of non-benzodiazepines (dexmedetomidine or propofol) with benzodiazepines (lorazepam or midazolam) [21]. Several reviews investigated different strategies to optimise sedation. For example, daily sedation interruption, protocolized sedation and comparisons of different depths of sedation [29, 35, 57].

### ICU delirium outcome mapping

Thirteen reviews were included in mapping delirium outcomes (Table 4; effect estimates in Additional file 4) [6, 7, 20, 22, 23, 27, 35, 36, 45, 52, 57–59]. Evidence for reducing delirium occurrence was mostly favourable for the α2-adrenoceptor agonist class (primarily dexmedetomidine) as compared with placebo (moderate to high-certainty evidence; 2 systematic reviews) [7, 23] or other intervention (low to high certainty; 4 systematic reviews) [7, 22, 36, 58]. However, the evidence was very uncertain in one comparison of dexmedetomidine versus mixed sedation types [52], and there may have been little or no difference between dexmedetomidine and mixed sedation types in another review [59]. All other pharmacological interventions had low- to high-certainty evidence of little or no difference in delirium occurrence compared with placebo or another intervention (3 systematic reviews) [7, 45, 57] or the evidence was very uncertain (one systematic review) [27]. Fewer reviews reported delirium duration and severity; these had moderate- to high-certainty evidence of little or no difference between interventions (one systematic review) [6] or the evidence was very uncertain (two systematic reviews) [36, 52]. No review compared (modified) intention-to-treat with available case or as-treated analyses.

**Table 4:**
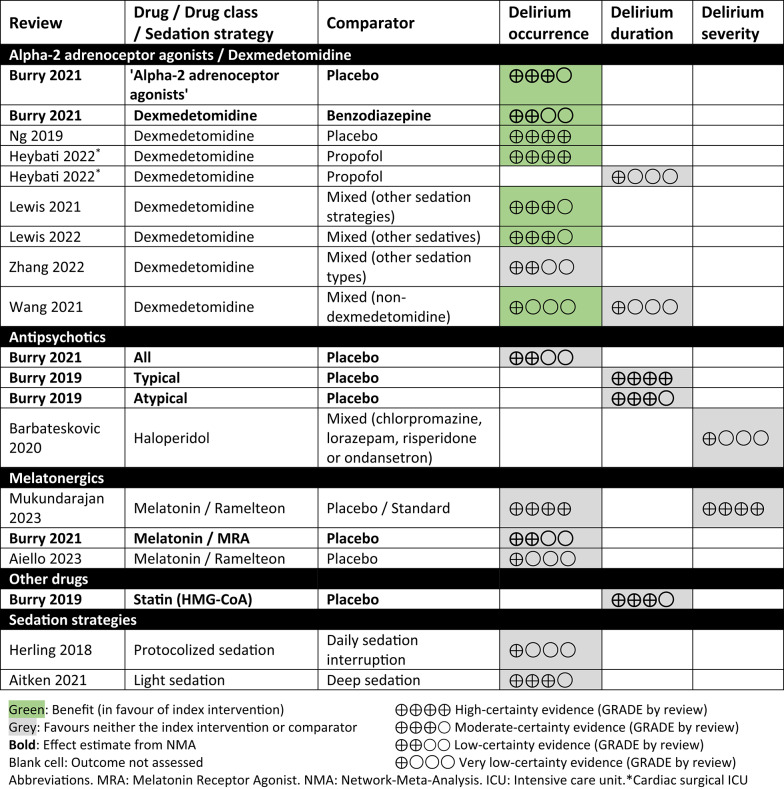
Effects of pharmacological interventions on delirium outcomes (pooled analyses of direct comparisons with GRADE assessment)

### ICU and hospital mortality outcome mapping

Six included reviews reported analyses with GRADE assessment for ICU and/or hospital mortality (Table 5; effect estimates in Additional file 5) [21, 29, 35, 45, 52, 57]. The results indicated little or no difference between any pharmacological intervention for ICU mortality (low- to high-certainty evidence; 4 systematic reviews) [29, 45, 52, 57]; for hospital mortality, there was moderate-certainty evidence of little or no difference between interventions (three systematic reviews) [21, 35, 57] or the evidence was very uncertain for dexmedetomidine versus non-dexmedetomidine (one systematic review) [52].

**Table 5:**
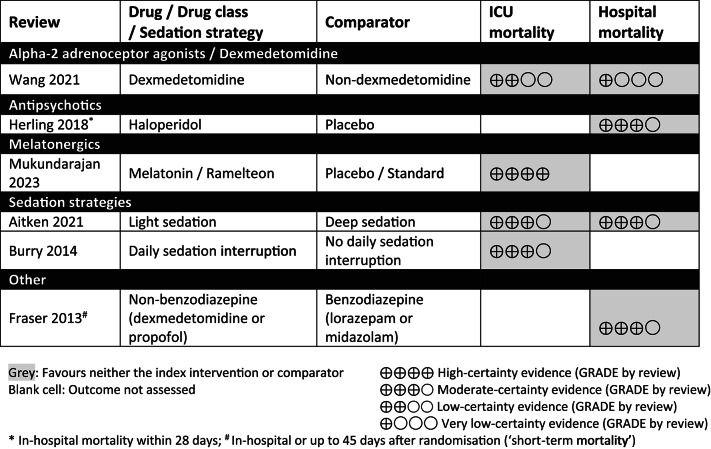
Effects of pharmacological interventions on ICU and hospital mortality (pooled analyses of direct comparisons with GRADE assessment)

### ICU and hospital length of stay mapping

Fifteen reviews were included in mapping ICU or hospital length of stay (Table 6; effect estimates in Additional file 6) [6, 7, 21, 22, 27, 29, 30, 35, 36, 45, 52, 57–60]. Effect estimates from four systematic reviews were favourable for α2-adrenoceptor agonists (primarily dexmedetomidine) reducing ICU length of stay compared to placebo (moderate-certainty evidence; one systematic review) [7] or other intervention (low- to moderate-certainty evidence) [21, 22, 58] although the evidence was very uncertain in several systematic reviews, and there was high-certainty evidence of no difference in ICU length of stay in one comparison of dexmedetomidine versus other, mixed sedatives [59]. There was also low- to high-certainty evidence (six systematic reviews) [6, 7, 29, 35, 45, 57] of little or no difference for ICU length of stay between other pharmacological comparisons or the evidence was very uncertain (one systematic review) [27]. However, remifentanil may have reduced ICU length of stay compared to other opioids (low-certainty evidence) [60]. There was very uncertain evidence for reduced hospital length of stay in one comparison of dexmedetomidine versus non-dexmedetomidine intervention [52]. In one review, an α2-adrenoceptor agonist class probably reduced hospital length of stay compared to placebo (moderate certainty) [7] but there was probably little or no difference between dexmedetomidine and other sedatives in another review (moderate certainty) [22]. The results for other pharmacological comparisons suggested low- to moderate-certainty evidence of little or no difference in hospital length of stay or the evidence was very uncertain.

**Table 6:**
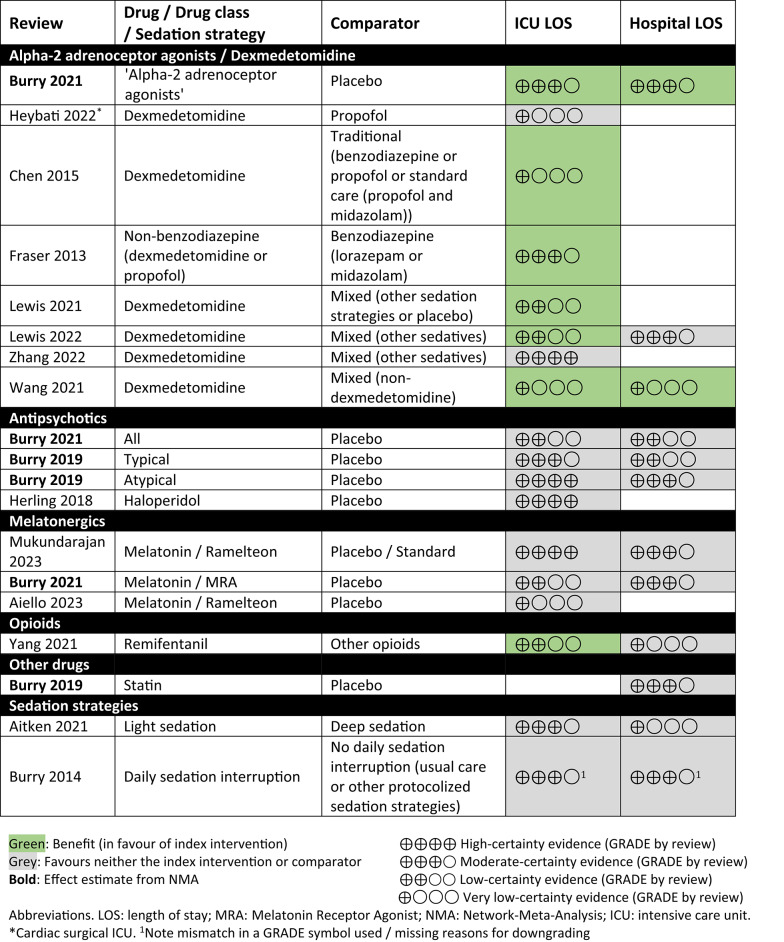
Effects of pharmacological interventions on ICU and hospital length of stay (pooled analyses of direct comparisons with GRADE assessment)

### Quality of the mapped evidence for delirium outcomes

Where reported, the reasons for downgrading the certainty of evidence in reviews of dexmedetomidine included imprecision, inconsistency, risk of bias and publication bias [7, 36, 52]. Evidence for all other pharmacological interventions was downgraded for reasons including imprecision, inconsistency and indirectness [6, 7, 27, 35].

### Network Meta-Analyses (NMA)

*Delirium occurrence:* Three NMAs reported delirium occurrence [7, 10, 25]. One NMA reported GRADE assessment [7]. This NMA considered direct and indirect evidence and found moderate-certainty evidence of the α2-adrenoceptor agonist drug class (primarily dexmedetomidine) probably reducing delirium occurrence compared to placebo and low-certainty evidence in favour of dexmedetomidine compared to benzodiazepines, while other comparisons were too uncertain to draw conclusions [7].

*Duration of delirium:* Two NMAs reported duration of delirium [5, 6]. One NMA reported GRADE assessment [6] and found the 95% credible interval was wide and included the possibility of little or no difference for all pharmacological interventions, including evidence from one study of dexmedetomidine, compared to placebo (low- to moderate-certainty evidence).

*Delirium severity:* There were no complete network maps reported for delirium severity.

### Other core outcomes: quality of life, cognition and emotional distress

One review performed pairwise meta-analysis of health-related quality of life at six months or more post-discharge. The review population involved ICU survivors who had been critically ill and required invasive mechanical ventilation [29]. They received either daily sedation interruption or no daily sedation interruption. Results included the possibility of an effect in either direction; there was no GRADE assessment of the certainty of the evidence. No meta-analyses were found for endpoint or change outcomes in cognition or emotional distress within ICU and/or post-discharge.

### Adverse event mapping

Types of adverse events reported in reviews of pharmacological interventions are shown in Additional file 7. These were identified from across all included reviews in the meta-review (with/without meta-analysis). They were classified broadly as any type of adverse event, and subtypes as haemodynamic, cardiac, extrapyramidal or other. Heart rate and blood pressure were among those measures more commonly selected for statistical analysis. However, the evidence highlighted variation in the interpretation of continuous measures as adverse outcomes or not. A minority of included reviews recorded delirium within adverse outcome reporting (see Additional file 3b).

## Discussion

We mapped outcomes in the Del-CorS core outcome set, as well as length of stay in the ICU or hospital for 17 of 56 included systematic reviews. The use of an α2-adrenoceptor agonist (primarily dexmedetomidine) had the largest evidence base and was mostly favourable over placebo and other pharmacological interventions for preventing or reducing delirium occurrence and ICU length of stay. There was no evidence to support any pharmacological intervention reducing ICU mortality.

The impact of loss to follow-up, including death, on delirium outcomes is unclear; no review compared intention-to-treat with available case or as-treated analyses for delirium occurrence. Unmapped reviews mostly pooled multiple timepoints (ICU and any) for mortality. Methodological limitations included incomplete reporting of individual agents as part of complex interventions, and their potential for effect modification, not reporting units of measurement for some outcomes (e.g. length of stay in days or hours), and variable approaches to meta-analysis and data transformation (e.g. median to mean number of days for length of stay). There was also variation in how some outcomes were conceptualised, for example delirium severity was measured using both duration (time measurement) and different numerical rating scales. No review reported outcomes for cognition, emotional distress, or change in quality-of-life although one review estimate included the possibility of little or no difference between sedation strategies for health-related quality of life among ICU survivors.

We applied an internationally agreed core outcome set to evaluate ICU delirium [18] but found variation in the meaning and interpretation of ICU delirium, such that it was also sometimes unclear whether the intervention was directed at prevention or management. We focused on meta-analysed evidence (primarily in ICU and hospital) although at least one narrative review with planned meta-analysis decided against pooling due to the heterogeneity of the population, interventions, comparators and outcomes [46]. We had to amend the protocol and proposed mapping strategy because reviews reported the study setting and some interventions differently; for example, study settings were not always reported as “ICU” and sedation strategies were reported as pharmacological interventions or multicomponent bundles. One excluded review [61] addressed non-pharmacological interventions and included two studies focused on protocolized sedation or awakening and breathing although neither intervention was meta-analysed. It was beyond the current meta-review to explore all potentially relevant subgroup and sensitivity analyses (e.g. for subgroup populations, individual agents or drug dose). However, among the mixed critically ill population, approximately 30% of included reviews focused on mechanically ventilated patients only.

An earlier overview of pharmacological interventions [2] found several hundred narrative reviews but only one systematic review and eight partially systematic reviews. Findings were broadly consistent with our meta-review although they found reviews classified only as prevention not management and did not map the certainty of the evidence across core outcomes. The overview did not report potential effects of interventions on outcomes including delirium severity, emotional distress, and ICU and hospital length of stay, and there was a lack of synthesised evidence found on quality-of-life and cognitive function.

One of the challenges for outcome reporting in this meta-review is that most primary studies of ICU delirium do not collect baseline data at ICU admission to evaluate change in ICU delirium and other core outcomes. In a clinically heterogenous population, this missing information could confound our understanding of causal relationships between interventions and outcomes.

Specific subgroups, such as patients with chronic pain or common psychiatric disorders, might benefit from pharmacological interventions that are not indicated for the general ICU population. However, our meta-review suggests that patients with psychiatric disorders are widely excluded from trials, so the benefits or harms of pharmacological intervention in this population are unknown. While some care bundles attempt to integrate the assessment, identification and management of pain, our meta-review has shown inconsistent use of rescue treatment, such as pain medication, that is not fully captured by the analysis of intervention effect. Our meta-review also highlights variation in how interventions are classified and that, despite greater confidence in the use of dexmedetomidine (and sparse evidence for other α2-adrenoceptor agonist drugs), the certainty of evidence is judged differently by different evidence syntheses (i.e. research teams) and across different core outcomes.

Our findings did not uncover possible variations in implementation or drug response by patient age, comorbidity and pharmacokinetics, such as potential hepatic impairment and interaction effects with other medications. Dexmedetomidine has been associated with an increased risk of mortality among ventilated critically ill patients under 65 years, possibly related to different infusion rates [62], although the mechanism remains uncertain. Dexmedetomidine causes limited respiratory depression and less impairment of consciousness compared to other sedatives (e.g. propofol). This could allow for better patient engagement in assessment of delirium (delirium cannot be diagnosed in coma/deep anaesthesia), and therefore some bias may exist if delirium alone is used as an outcome (i.e. not considering delirium and coma together).

Our meta-review highlighted the possibility of publication bias, emphasising the importance of independent research into interventions for ICU delirium. More research is needed that controls for the baseline population admitted to an ICU, addressing the underlying conditions indicating or precluding intervention, pharmacokinetics, and how these relate to core ICU outcomes that are important to patients. Baseline assessment at study randomisation may be feasible within a subset of patients who are risk-stratified (e.g. for age and comorbidities), and loss to follow-up investigated to support further analysis.

## Conclusions

This meta-review has shown that the α2-adrenoceptor agonist drug class (primarily dexmedetomidine) had the largest evidence base of the pharmacological interventions and is probably favourable over placebo for preventing/reducing the occurrence of ICU delirium. However, the evidence base for this ranged from very uncertain to high certainty in favour of dexmedetomidine compared to other interventions. The evidence across ICU and hospital length of stay outcomes was variable for dexmedetomidine. There may have been little or no difference between dexmedetomidine and a non-dexmedetomidine comparator for ICU mortality and the evidence was too uncertain to draw conclusions on hospital mortality. We found the distinction between prevention and treatment/management was sometimes unclear in reviews and there was a lack of certainty or assessment of the certainty of the evidence to support the use of interventions other than dexmedetomidine (including sedation strategies) for either prevention or treatment/management of ICU delirium. We found no evidence for the effects of pharmacological interventions on either follow-up or change in cognition and emotional distress. As the evidence remains unclear on an optimal combination of interventions for preventing or managing ICU delirium, there is a critical need for a well-designed RCT testing a multicomponent intervention that includes the most promising elements identified in this review, as well as those identified in the non-pharmacological review (Part 2 [63]). This type of rigorous evaluation would establish a stronger evidence base for clinical practice guidelines.

## Supplementary Information

Additional file 1: Databases, search results and search strings.

Additional file 2a: Data extraction template.

Additional file 2b: Exclusion reasons and references.

Additional file 3a: Included review characteristics - population.

Additional file 3b: Included review characteristics - interventions, comparators and outcomes.

Additional file 4: Pharmacological effect estimates for mapped ICU delirium outcomes.

Additional file 5: Pharmacological effect estimates for mapped ICU and hospital mortality outcomes.

Additional file 6: Pharmacological effect estimates for mapped ICU and hospital length of stay outcomes.

Additional file 7: Types of adverse events reported in pharmacological reviews of ICU delirium.

Additional file 8: PRISMA ScR Checklist.

Additional file 9: PRIOR Checklist.

## Data Availability

No datasets were generated or analysed during the current study.

## Abbreviations

CAM: Confusion Assessment Method
CAM-ICU: Confusion Assessment Method for the ICU
Del-COrS: Development of a Core Outcome Set for effectiveness trials of interventions to prevent and/or treat delirium
DSM: Diagnostic and Statistical Manual for mental disorders
GRADE: Grading of Recommendations, Assessment, Development, and Evaluations
HDU: High Dependency care Unit
ICDSC: Intensive Care Delirium Screening Checklist
ICU: Intensive Care Unit
LOS: Length Of Stay
MA: Meta-Analysis
MRA: Melatonin Receptor Agonist
NMA: Network Meta-Analysis
POD: Post-Operative Delirium
PRIOR: Preferred Reporting Items for Overviews of Reviews
PRISMA: Preferred Reporting Items for Systematic Reviews and Meta-Analyses
PRISMA-ScR: Preferred Reporting Items for Systematic reviews and Meta-Analyses extension for Scoping Reviews
RCT: Randomised Controlled Trial
SR: Systematic Review
