# Supplementary material for: A systematic meta-review of interventions to prevent and manage delirium in the Intensive Care Unit: Part 1 – Pharmacological interventions
Source: Crit Care. 2025 Dec 30;29:540. doi: 10.1186/s13054-025-05615-0 (PMC12751364; doi:10.1186/s13054-025-05615-0)
Supplement: Supplementary file 1 — Additional file 1: Databases, search results and search strings. [file 13054_2025_5615_MOESM1_ESM.docx]

**Additional file 1: Databases, search results and search strings**

Epistemonikos (searched 19 July 2023)

265 reviews; 16 broad syntheses

(intensive care units OR critical care units or Critical illness) AND (delirium OR delir*)

**Table of Databases and Search Results**

| **Database** | **Dates searched** | **Number of references retrieved** | **Number after de-duplication** |
| --- | --- | --- | --- |
| **MEDLINE (Ovid)**  and  Ovid MEDLINE(R) In-Process & Other Non-Indexed Citations  $$ Medline | 1946 to September 26, 2023 | 528 |  |
| **EMBASE (Ovid)**  $$Embase | 1974 to 2023 Week 38 | 977 |  |
| **Cochrane Database of Systematic Reviews**  $$CDSR | Searched 28/09/2023 | 18 |  |
| **CINAHL (EBSCOHost)**  $$CINAHL | 1981 to September 28, 2023 | 437 |  |
| **PsycINFO (Ovid)**  $$PsycINFO | 1806 to Week 3, 2023 | 86 |  |
| **Scopus**  $$Scopus | 1823 to September 28, 2023 | 506 |  |
| **Web of Science**  $$WoS | 1900 to September 28, 2023 | 829 |  |
| **All databases** |  | **3,381** |  |

**MEDLINE**

**1**  exp confusion/
**2**  deliri*.ti,ab.
**3**  (acute adj2 (confusion* or "brain syndrome" or "brain failure" or "psycho-organic syndrome" or "organic psychosyndrome" or "organic brain syndrome")).ti,ab.
**4**  (terminal* adj restless*).ti,ab.
**5**  (toxic adj2 (confus$ or psychosis)).ti,ab.
**6**  metabolic encephalopathy.ti,ab.
**7**  clouded state.ti,ab.
**8**  "clouding of consciousness".ti,ab.
**9**  exogenous psychosis.ti,ab.
**10**  or/1-9
**11**  exp Intensive Care Units/
**12**  Intensive Care.ti,ab.
**13**  ICU.ti,ab.
**14**  Critical care/
**15**  (Critical adj2 (care or ill or illness*)).ti,ab.
**16**  (high dependency unit* or HDU).ti,ab.
**17**  or/11-16
**18**  (systematic review or meta-analysis).pt.
**19**  meta-analysis/ or systematic review/ or systematic reviews as topic/ or meta-analysis as topic/ or exp technology assessment, biomedical/ or network meta-analysis/
**20**  ((systematic* adj3 (review* or overview*)) or (methodologic* adj3 (review* or overview*))).ti,ab,kf. (331263)
**21**  ((quantitative adj3 (review* or overview* or synthes*)) or (research adj3 (integrati* or overview*))).ti,ab,kf.
**22**  ((integrative adj3 (review* or overview*)) or (collaborative adj3 (review* or overview*)) or (pool* adj3 analy*)).ti,ab,kf.
**23**  (data synthes* or data extraction* or data abstraction*).ti,ab,kf.
**24**  (handsearch* or hand search*).ti,ab,kf.
**25**  (mantel haenszel or peto or der simonian or dersimonian or fixed effect* or latin square*).ti,ab,kf.
**26**  (met analy* or metanaly* or technology assessment* or HTA or HTAs or technology overview* or technology appraisal*).ti,ab,kf.
**27**  (meta regression* or metaregression*).ti,ab,kf.
**28**  (meta-analy* or metaanaly* or systematic review* or biomedical technology assessment* or bio-medical technology assessment*).mp,hw.
**29**  (medline or cochrane or pubmed or medlars or embase or cinahl).ti,ab,hw.
**30**  (cochrane or (health adj2 technology assessment) or evidence report).jw.
**31**  (comparative adj3 (efficacy or effectiveness)).ti,ab,kf.
**32**  (outcomes research or relative effectiveness).ti,ab,kf.
**33**  ((indirect or indirect treatment or mixed-treatment or bayesian) adj3 comparison*).ti,ab,kf.
**34**  (multi* adj3 treatment adj3 comparison*).ti,ab,kf.
**35**  (mixed adj3 treatment adj3 (meta-analy* or metaanaly*)).ti,ab,kf.
**36**  umbrella review*.ti,ab,kf.
**37**  (multi* adj2 paramet* adj2 evidence adj2 synthesis).ti,ab,kf.
**38**  (multiparamet* adj2 evidence adj2 synthesis).ti,ab,kf.
**39**  (multi-paramet* adj2 evidence adj2 synthesis).ti,ab,kf.
**40**  or/18-39
**41**  and/10,17,40

*Lines 18 to 39 are taken from the [CADTH SR / MA / HTA / ITC - MEDLINE, Embase, PsycInfo](https://searchfilters.cadth.ca/list?q=&ps=20&topic_facet=health%20technology%20assessments%20000000%7CHealth%20technology%20assessments&p=1&name_facet=medline%20000000%7CMEDLINE) search filter, adapted for Ovid Medline

**Embase**

**1**  exp delirium/
**2**  exp confusion/
**3**  deliri*.ti,ab.
**4**  (acute adj2 (confusion* or "brain syndrome" or "brain failure" or "psycho-organic syndrome" or "organic psychosyndrome" or "organic brain syndrome")).ti,ab.
**5**  (terminal* adj restless*).ti,ab.
**6**  (toxic adj2 (confus* or psychosis)).ti,ab.
**7**  metabolic encephalopathy.ti,ab.
**8**  clouded state.ti,ab.
**9**  "clouding of consciousness".ti,ab.
**10**  exogenous psychosis.ti,ab.
**11**  or/1-10
**12**  exp intensive care unit/
**13**  Intensive Care.ti,ab.
**14**  ICU.ti,ab.
**15**  exp intensive care/
**16**  (Critical adj2 (care or ill or illness*)).ti,ab.
**17**  (high dependency unit* or HDU).ti,ab.
**18**  or/12-17
**19**  meta-analysis/
**20**  systematic review/
**21**  systematic reviews as topic/
**22**  meta-analysis as topic/
**23**  exp technology assessment, biomedical/
**24**  network meta-analysis/
**25**  ((systematic* adj3 (review* or overview*)) or (methodologic* adj3 (review* or overview*))).ti,ab,kf.
**26**  ((quantitative adj3 (review* or overview* or synthes*)) or (research adj3 (integrati* or overview*))).ti,ab,kf.
**27**  ((integrative adj3 (review* or overview*)) or (collaborative adj3 (review* or overview*)) or (pool* adj3 analy*)).ti,ab,kf.
**28**  (data synthes* or data extraction* or data abstraction*).ti,ab,kf.
**29**  (handsearch* or hand search*).ti,ab,kf.
**30**  (mantel haenszel or peto or der simonian or dersimonian or fixed effect* or latin square*).ti,ab,kf.
**31**  (met analy* or metanaly* or technology assessment* or HTA or HTAs or technology overview* or technology appraisal*).ti,ab,kf.
**32**  (meta regression* or metaregression*).ti,ab,kf.
**33**  (meta-analy* or metaanaly* or systematic review* or biomedical technology assessment* or bio-medical technology assessment*).mp,hw.
**34**  (medline or cochrane or pubmed or medlars or embase or cinahl).ti,ab,hw.
**35**  (cochrane or (health adj2 technology assessment) or evidence report).jw.
**36**  (comparative adj3 (efficacy or effectiveness)).ti,ab,kf.
**37**  (outcomes research or relative effectiveness).ti,ab,kf.
**38**  ((indirect or indirect treatment or mixed-treatment or bayesian) adj3 comparison*).ti,ab,kf.
**39**  (multi* adj3 treatment adj3 comparison*).ti,ab,kf.
**40**  (mixed adj3 treatment adj3 (meta-analy* or metaanaly*)).ti,ab,kf.
**41**  umbrella review*.ti,ab,kf.
**42**  (multi* adj2 paramet* adj2 evidence adj2 synthesis).ti,ab,kf.
**43**  (multiparamet* adj2 evidence adj2 synthesis).ti,ab,kf.
**44**  (multi-paramet* adj2 evidence adj2 synthesis).ti,ab,kf.
**45**  or/19-44
**46**  and/11,18,45
**47**  limit 46 to (article or article in press or "review")

*Lines 19 to 44 are taken from the [CADTH SR / MA / HTA / ITC - MEDLINE, Embase, PsycInfo](https://searchfilters.cadth.ca/list?q=&ps=20&topic_facet=health%20technology%20assessments%20000000%7CHealth%20technology%20assessments&p=1&name_facet=medline%20000000%7CMEDLINE) search filter, adapted for Ovid Embase

**Cochrane Database of Systematic Reviews**

#1 MeSH descriptor: [Confusion] explode all trees

#2 deliri*:ti,ab

#3 (acute NEAR/2 (confusion* or "brain syndrome" or "brain failure" or "psycho-organic syndrome" or "organic psychosyndrome" or "organic brain syndrome")):ti,ab

#4 (terminal* NEXT restless*):ti,ab

#5 (toxic NEAR/2 (confus* or psychosis)):ti,ab

#6 "metabolic encephalopathy":ti,ab

#7 "clouded state":ti,ab

#8 "clouding of consciousness":ti,ab

#9 "exogenous psychosis":ti,ab

#10 #1 OR #2 OR #3 OR #4 OR #5 OR #6 OR #7 OR #8 OR #9

#11 MeSH descriptor: [Intensive Care Units] explode all trees

#12 "Intensive Care":ti,ab

#13 ICU:ti,ab

#14 MeSH descriptor: [Critical Care] explode all trees

#15 (Critical NEAR/2 (care or ill or illness*)):ti,ab

#16 ("high dependency unit" OR "high dependency units" OR HDU):ti,ab

#17 #11 OR #12 OR #13 OR #14 OR #15 OR #16

#18 #10 AND #17

**CINAHL**

S1 (MH "Confusion+")

S2 TI deliri* OR AB deliri*

S3 TI ( (acute N2 (confusion* or "brain syndrome" or "brain failure" or "psycho-organic syndrome" or "organic psychosyndrome" or "organic brain syndrome")) ) OR AB ( (acute N2 (confusion* or "brain syndrome" or "brain failure" or "psycho-organic syndrome" or "organic psychosyndrome" or "organic brain syndrome")) )

S4 TI "terminal* restless*" OR AB "terminal* restless*"

S5 TI ( (toxic N2 (confus* or psychosis)) ) OR AB ( (toxic N2 (confus* or psychosis)) )

S6 TI "metabolic encephalopathy" OR AB "metabolic encephalopathy"

S7 TI "clouded state" OR AB "clouded state"

S8 TI "clouding of consciousness" OR AB "clouding of consciousness"

S9 TI "exogenous psychosis" OR AB "exogenous psychosis"

S10 S1 OR S2 OR S3 OR S4 OR S5 OR S6 OR S7 OR S8 OR S9

S11 (MH "Intensive Care Units+")

S12 TI "Intensive Care" OR AB "Intensive Care"

S13 TI ICU OR AB ICU

S14 (MH "Critical Care+")

S15 TI ( (Critical N2 (care or ill or illness*)) ) OR AB ( (Critical N2 (care or ill or illness*)) )

S16 TI ( ("high dependency unit" OR "high dependency units" OR HDU) ) OR AB ( ("high dependency unit" OR "high dependency units" OR HDU) )

S17 S11 OR S12 OR S13 OR S14 OR S15 OR S16

S18 MH "meta analysis"

S19 MH "systematic review"

S20 MH "Technology, Medical/EV"

S21 PT "systematic review"

S22 PT "meta analysis"

S23 (((TI systematic* OR AB systematic*) N3 ((TI review* OR AB review*) OR (TI overview* OR AB overview*))) OR ((TI methodologic* OR AB methodologic*) N3 ((TI review* OR AB review*) OR (TI overview* OR AB overview*)))

S24 (((TI quantitative OR AB quantitative) N3 ((TI review* OR AB review*) OR (TI overview* OR AB overview*) OR (TI synthes* OR AB synthes*))) OR ((TI research OR AB research) N3 ((TI integrati* OR AB integrati*) OR (TI overview* OR AB overview*)))

S25 (((TI integrative OR AB integrative) N3 ((TI review* OR AB review*) OR (TI overview* OR AB overview*)))

S26 ((TI collaborative OR AB collaborative) N3 ((TI review* OR AB review*) OR (TI overview* OR AB overview*)))

S27 ((TI pool* OR AB pool*) N3 (TI analy* OR AB analy*)))

S28 ((TI "data synthes*" OR AB "data synthes*")

S29 (TI "data extraction*" OR AB "data extraction*")

S30 (TI "data abstraction*" OR AB "data abstraction*"))

S31 ((TI handsearch* OR AB handsearch*)

S32 (TI "hand search*" OR AB "hand search*"))

S33 ((TI "mantel haenszel" OR AB "mantel haenszel")

S34 (TI peto OR AB peto)

S35 (TI "der simonian" OR AB "der simonian")

S36 (TI dersimonian OR AB dersimonian)

S37 (TI "fixed effect*" OR AB "fixed effect*")

S38 (TI "latin square*" OR AB "latin square*"))

S39 (TI "met analy*" OR AB "met analy*")

S40 (TI metanaly* OR AB metanaly*)

S41 (TI "technology assessment*" OR AB "technology assessment*")

S42 (ΤΙ ΗΤΑ ΟΡ ΑΒ ΗΤΑ)

S43 (TI HTAs OR AB HTAs)

S44 (TI "technology overview*" OR AB "technology overview*")

S45 (TI "technology appraisal*" OR AB "technology appraisal*"))

S46 ((TI "meta regression*" OR AB "meta regression*")

S47 (TI metaregression* OR AB metaregression*))

S48 (TI meta-analy* OR TI metaanaly* OR TI "systematic review*" OR TI "biomedical technology assessment*" OR TI "bio-medical technology assessment*" OR AB meta-analy* OR AB metaanaly* OR AB "systematic review*" OR AB "biomedical technology assessment*" OR AB "bio-medical technology assessment*" OR MW meta-analy* OR MW metaanaly* OR MW "systematic review*" OR MW "biomedical technology assessment*" OR MW "bio-medical technology assessment*")

S49 (TI medline OR AB medline OR MW medline)

S50 (TI cochrane OR AB cochrane OR MW cochrane)

S51 (TI pubmed OR AB pubmed OR MW pubmed)

S52 (TI medlars OR AB medlars OR MW medlars)

S53 (TI embase OR AB embase OR MW embase)

S54 (TI cinahl OR AB cinahl OR MW cinahl)

S55 (SO Cochrane OR SO health technology assessment OR SO evidence report)

S56 ((TI comparative OR AB comparative) N3 ((TI efficacy OR AB efficacy))

S57 (TI effectiveness OR AB effectiveness)

S58 (TI "outcomes research" OR AB "outcomes research")

S59 (TI "relative effectiveness" OR AB "relative effectiveness")

S60 (TI indirect OR AB indirect)

S61 (TI "indirect treatment" OR AB "indirect treatment")

S62 (TI mixed-treatment OR AB mixed-treatment)

S63 ((TI bayesian OR AB bayesian)) N3 (TI comparison* OR AB comparison*))

S64 ((TI multi* OR AB multi*) N3 (TI treatment OR AB treatment) N3 (TI comparison* OR AB comparison*))

S65 ((TI mixed OR AB mixed) N3 (TI treatment OR AB treatment) N3 ((TI meta-analy* OR AB meta-analy*) OR (TI metaanaly* OR AB metaanaly*))

S66 TI "umbrella review*" OR AB "umbrella review*")

S67 ((TI multi* OR AB multi*) N2 (TI paramet* OR AB paramet*) N2 (TI evidence OR AB evidence) N2 (TI synthesis OR AB synthesis))

S68 ((TI multiparamet* OR AB multiparamet*) N2 (TI evidence OR AB evidence) N2 (TI synthesis OR AB synthesis))

S69 ((TI multi-paramet* OR AB multi-paramet*) N2 (TI evidence OR AB evidence) N2 (TI synthesis OR AB synthesis))

S70 S18 S19 OR S20 OR S21 OR S22 OR S23 OR S24 OR S25 OR S26 OR S27 OR S28 OR S29 OR S30 OR S31 OR S32 OR S33 OR S34 OR S35 OR S36 OR S37 OR S38 OR S39 OR S40 OR S41 OR S42 OR S43 OR S44 OR S45 OR S46 OR S47 OR S48 OR S49 OR S50 OR S51 OR S52 OR S53 OR S54 OR S55 OR S56 OR S57 OR S58 OR S59 OR S60 OR S61 OR S62 OR S63 OR S64 OR S65 OR S66 OR S67 OR S68 OR S69

S71 S10 AND S17 AND S70

### Lines S18 to S69 are taken from the [CADTH SR / MA / HTA / ITC – CINAHL search filter](https://searchfilters.cadth.ca/list?q=&p=1&ps=20&topic_facet=systematic%20reviews%20000000%7CSystematic%20reviews&name_facet=cinahl%20000000%7CCINAHL)

**PsycINFO**

**1** mental confusion/
**2**  delirium/
**3**  deliri*.ti,ab.
**4**  (acute adj2 (confusion* or "brain syndrome" or "brain failure" or "psycho-organic syndrome" or "organic psychosyndrome" or "organic brain syndrome")).ti,ab.
**5**  (terminal* adj restless*).ti,ab.
**6**  (toxic adj2 (confus$ or psychosis)).ti,ab.
**7**  metabolic encephalopathy.ti,ab.
**8**  clouded state.ti,ab.
**9**  "clouding of consciousness".ti,ab.
**10**  exogenous psychosis.ti,ab.
**11**  or/1-10
**12**  exp intensive care/
**13**  Intensive Care.ti,ab.
**14**  ICU.ti,ab.
**15**  (Critical adj2 (care or ill or illness*)).ti,ab.
**16**  (high dependency unit* or HDU).ti,ab.
**17**  or/12-16
**18**  "systematic review"/ or meta-analysis/ (6070)
**19**  ((systematic* adj3 (review* or overview*)) or (methodologic* adj3 (review* or overview*))).ti,ab.
**20**  ((quantitative adj3 (review* or overview* or synthes*)) or (research adj3 (integrati* or overview*))).ti,ab.
**21**  ((integrative adj3 (review* or overview*)) or (collaborative adj3 (review* or overview*)) or (pool* adj3 analy*)).ti,ab.
**22**  (data synthes* or data extraction* or data abstraction*).ti,ab.
**23**  (handsearch* or hand search*).ti,ab.
**24**  (mantel haenszel or peto or der simonian or dersimonian or fixed effect* or latin square*).ti,ab.
**25**  (met analy* or metanaly* or technology assessment* or HTA or HTAs or technology overview* or technology appraisal*).ti,ab.
**26**  (meta regression* or metaregression*).ti,ab.
**27**  (meta-analy* or metaanaly* or systematic review* or biomedical technology assessment* or bio-medical technology assessment*).mp,hw.
**28**  (medline or cochrane or pubmed or medlars or embase or cinahl).ti,ab,hw.
**29**  (cochrane or (health adj2 technology assessment) or evidence report).jw.
**30**  (comparative adj3 (efficacy or effectiveness)).ti,ab.
**31**  (outcomes research or relative effectiveness).ti,ab.
**32**  ((indirect or indirect treatment or mixed-treatment or bayesian) adj3 comparison*).ti,ab.
**33**  (meta-analysis or systematic review).md.
**34**  (multi* adj3 treatment adj3 comparison*).ti,ab.
**35**  (mixed adj3 treatment adj3 (meta-analy* or metaanaly*)).ti,ab.
**36**  umbrella review*.ti,ab.
**37**  (multi* adj2 paramet* adj2 evidence adj2 synthesis).ti,ab.
**38**  (multiparamet* adj2 evidence adj2 synthesis).ti,ab.
**39**  (multi-paramet* adj2 evidence adj2 synthesis).ti,ab.
**40**  or/18-39
**41**  and/11,17,40

*Lines 18 to 39 are taken from the [CADTH SR / MA / HTA / ITC - MEDLINE, Embase, PsycInfo](https://searchfilters.cadth.ca/list?q=&ps=20&topic_facet=health%20technology%20assessments%20000000%7CHealth%20technology%20assessments&p=1&name_facet=medline%20000000%7CMEDLINE) search filter, adapted for Ovid PsycINFO

**Scopus**

#1 TITLE-ABS-KEY ( deliri* )

#2 TITLE-ABS-KEY ( acute W/2 ( confusion* OR "brain syndrome" OR "brain failure" OR "psycho-organic syndrome" OR "organic psychosyndrome" OR "organic brain syndrome" )

#3 TITLE-ABS-KEY ( toxic W/2 ( confus* OR psychosis ) )

#4 TITLE-ABS-KEY ( "metabolic encephalopathy" )

#5 TITLE-ABS-KEY ( "clouded state" )

#6 TITLE-ABS-KEY ( "clouding of consciousness" )

#7 TITLE-ABS-KEY ( "exogenous psychosis" )

#8 #1 OR #2 OR #3 OR #4 OR #5 OR #6 OR #7

#9 TITLE-ABS-KEY ( "Intensive Care" )

#10 TITLE-ABS-KEY ( icu )

#11 TITLE-ABS-KEY ( ( critical W/2 ( care OR ill OR illness* ) )

#12 TITLE-ABS-KEY ( "high dependency unit*" OR hdu )

#13 #9 OR #10 OR #11 OR #12

#14 TITLE-ABS-KEY((systematic* W/3 (review* OR overview* )) OR (methodologic* W/3 (review* OR overview* )))

#15 TITLE-ABS-KEY((quantitative W/3 (review* OR overview* OR synthes* )) OR (research W/3 (integrati* OR overview* )))

#16 TITLE-ABS-KEY ((integrative W/3 (review* OR overview* )) OR (collaborative W/3 (review* OR overview* )) OR (pool* W/3 analy* ))

#17 TITLE-ABS-KEY("data synthes*" OR "data extraction*" OR "data abstraction*" )

#18 TITLE-ABS-KEY(handsearch* OR "hand search*" )

#19 TITLE-ABS-KEY("mantel haenszel" OR peto OR "der simonian" OR dersimonian OR "fixed effect*" OR "latin square*" )

#20 TITLE-ABS-KEY("met analy*" OR metanaly* OR "technology assessment*" OR HTA OR HTAs OR "technology overview*" OR "technology appraisal*" )

#21 TITLE-ABS-KEY("meta regression*" OR metaregression* )

#22 TITLE-ABS-KEY(meta-analy* OR metaanaly* OR "systematic review*" OR "biomedical technology assessment*" OR "bio-medical technology assessment*" )

#23 TITLE-ABS-KEY(medline OR cochrane OR pubmed OR medlars OR embase OR cinahl )

#24 SRCTITLE(cochrane OR (health W/2 "technology assessment" ) OR "evidence report" )

#25 TITLE-ABS-KEY(comparative W/3 (efficacy OR effectiveness ))

#26 TITLE-ABS-KEY("outcomes research" OR "relative effectiveness" )

#27 TITLE-ABS-KEY((indirect OR "indirect treatment" OR mixed-treatment OR bayesian ) W/3 comparison* )

#28 TITLE-ABS-KEY(multi* W/3 treatment W/3 comparison* )

#29 TITLE-ABS-KEY(mixed W/3 treatment W/3 (meta-analy* OR metaanaly* )) OR TITLE-ABS-KEY("umbrella review*")

#30 TITLE-ABS-KEY(multi* W/2 paramet* W/2 evidence W/2 synthesis )

#31 TITLE-ABS-KEY(multiparamet* W/2 evidence W/2 synthesis )

#32 TITLE-ABS-KEY(multi-paramet* W/2 evidence W/2 synthesis )

#33 #14 OR #15 OR #16 OR #17 OR #18 OR #19 OR #20 OR #21 OR #22 OR #23 OR #24 OR #25 OR #26 OR #27 OR #27 OR #29 OR #30 OR #31 OR #32

#34 #8 AND #13 AND #33

### *Lines 14 to 32 are taken from the CADTH [SR / MA / HTA / ITC – Scopus search filter](https://searchfilters.cadth.ca/list?q=&p=1&ps=20&topic_facet=systematic%20reviews%20000000%7CSystematic%20reviews&name_facet=scopus%20000000%7CScopus)

**Web of Science**

#1 TS=(deliri*)

#2 TS=((acute NEAR/2 (confusion* or "brain syndrome" or "brain failure" or "psycho-organic syndrome" or "organic psychosyndrome" or "organic brain syndrome")))

#3 TS=((toxic NEAR/2 (confus* or psychosis)))

#4 TS=("metabolic encephalopathy")

#5 TS=("clouded state" )

#6 TS=("clouding of consciousness")

#7 TS=("exogenous psychosis" )

#8 #7 OR #6 OR #5 OR #4 OR #3 OR #2 OR #1

#9 TS=("Intensive Care")

#10 TS=(ICU)

#11 TS=((Critical NEAR/2 (care or ill or illness*)))

#12 TS=("high dependency unit*" OR HDU)

#13 #9 OR #10 OR #11 OR #12

#14 TS=(systematic* NEAR/3 (review* OR overview* ))

#15 TS=(methodologic* NEAR/3 (review* OR overview* ))

#16 TS=(quantitative NEAR/3 (review* OR overview* OR synthes* ))

#17 TS=(research NEAR/3 (integrati* OR overview* ))

#18 TS=(integrative NEAR/3 (review* OR overview* ))

#19 TS=(collaborative NEAR/3 (review* OR overview* ))

#20 TS=(pool* NEAR/3 analy* )

#21 TS=("data synthes*" OR "data extraction*" OR "data abstraction*" )

#22 TS=(handsearch* OR "hand search*" )

#23 TS=("mantel haenszel" OR peto OR "der simonian" OR dersimonian OR "fixed effect*" OR "latin square*" )

#24 TS=("met analy*" OR metanaly* OR "technology assessment*" OR HTA OR HTAs OR "technology overview*" OR "technology appraisal*" )

#25 TS=("meta regression*" OR metaregression* )

#26 TS=(meta-analy* OR metaanaly* OR "systematic review*" OR "biomedical technology assessment*" OR "bio-medical technology assessment*" )

#27 TS=(medline OR cochrane OR pubmed OR medlars OR embase OR cinahl )

#28 SO=(COCHRANE DATABASE OF SYSTEMATIC REVIEWS)

#29 TS=(comparative NEAR/3 (efficacy OR effectiveness ) )

#30 TS=("outcomes research" OR "relative effectiveness" )

#31 TS=((indirect OR "indirect treatment" OR mixed-treatment OR bayesian) NEAR/3 comparison* )

#32 TS=(multi* NEAR/3 treatment NEAR/3 comparison* )

#33 TS=((mixed NEAR/3 treatment NEAR/3 (meta-analy* OR metaanaly* )) )

#34 TS=("umbrella review*")

#35 TS=((multi* NEAR/2 paramet* NEAR/2 evidence NEAR/2 synthesis ) )

#36 TS=((multiparamet* NEAR/2 evidence NEAR/2 synthesis ) )

#37 TS=((multi-paramet* NEAR/2 evidence NEAR/2 synthesis ))

#38 #24 OR #23 OR #22 OR #21 OR #20 OR #19 OR #18 OR #17 OR #16 OR #15 OR #14 OR #13 OR #12 OR #11 OR #10 OR #9 OR #8 OR #7 OR #6 OR #5 OR #4 OR #3 OR #2 OR #1

#39 #8 AND #13 AND #38

*Lines 14 to 37 are taken from the CADTH [SR / MA / HTA / ITC – Scopus search filter](https://searchfilters.cadth.ca/list?q=&p=1&ps=20&topic_facet=systematic%20reviews%20000000%7CSystematic%20reviews&name_facet=scopus%20000000%7CScopus) adapted for Web of Science
