# Supplementary material for: A systematic meta-review of interventions to prevent and manage delirium in the Intensive Care Unit: Part 1 – Pharmacological interventions
Source: Crit Care. 2025 Dec 30;29:540. doi: 10.1186/s13054-025-05615-0 (PMC12751364; doi:10.1186/s13054-025-05615-0)
Supplement: Supplementary file 2 — Additional file 2a: Data extraction template. [file 13054_2025_5615_MOESM2_ESM.docx]

**Additional file 2a: Data extraction template**

| Section of review | Review | First author (year) |
| --- | --- | --- |
| Title page | Publishing journal | Name of journal |
| Title | Full title of review | Full title |
| Methods | Review type | Systematic, scoping, narrative, other from Methods |
|  | Included study design(s) | RCT, randomised trials, controlled trials, cohort studies, quasi-experimental, other from Methods |
|  | Included cluster RCTs | Yes/No or Not reported - cluster RCTs identified in the Methods eligibility criteria |
|  | Inclusion criteria | Verbatim as reported in Methods |
|  | Exclusion criteria | Verbatim as reported in Methods |
|  | Stratum | Critical illness (ICU), mechanical ventilation, trauma, post-surgery from Methods eligibility criteria |
| Methods/Results /Abstract | Total number of included studies | n=xx (in PRISMA flow diagram) [If no PRISMA flow diagram report n=xx (in Results) or n=xx (in Abstract) or Not reported] |
| Methods/Results | Proportion of studies in ICU setting | 80-100% (n=xx) [From Results if ICU setting not in Eligibility criteria] |
| Results | Country | Country (n=xx) [From Results] |
|  | Method of assessment of meta-review condition | Screening or diagnostic tools for ICU delirium from Results |
|  | Method of assessment of any other diagnoses | Screening or diagnostic tools e.g. for dementia, PTSD or other psychiatric condition from Results |
|  | Clinical population | Age, clinical condition from Results |
|  | Additional population information | Gender and ethnicity from Results |
|  | Number of included pharmacological RCTs (number of participants) | n=xx [Single and combination - Add manually from Results if not reported] or Not applicable |
|  | Number of included non-pharmacological RCTs (number of participants) | n=xx [Single and combination - Add manually from Results if not reported] or Not applicable |
|  | Number of included multicomponent RCTs (number of participants) | n=xx [Add manually from Results if not reported] or Not applicable |
|  | Number of included non-pharmacological non-RCTs (number of participants) | n=xx [Add manually from Results if not reported] or Not applicable |
|  | Number of included multicomponent non-RCTs (number of participants) | n=xx [Add manually from Results if not reported] or Not applicable |
|  | Proportion of relevant included studies published from 2000 onwards | 0-100% (n=xx) [Add manually from Results for 'pharmacological' or 'non-pharmacological' studies] |
|  | Review Intervention(s) | Name(s) of intervention from included study characteristics in Results (any further study-level description, e.g., drug class, dose range and mode of delivery) [List all interventions except placebo, usual care or no treatment here] |
|  | Placebo/Usual care/No treatment | Specify Placebo, Usual care or No treatment here from included study characteristics in Results (any further study-level description e.g., dose range and mode of delivery) |
| Methods/Results/ Discussion | Concurrent intervention(s) | Reported Yes/No; Name(s) of any other concurrent intervention not investigated from Methods or Results or Discussion (any further study-level or review-level description e.g., drug class, dose range and mode of delivery) |
| Results/Discussion | Unit of analysis issues | Yes/No or Not reported in Results or Discussion; for example, meta-analysis of cluster and individual RCTs |
| Methods/Results | Risk of bias assessment | Yes/No from Methods/Results; Name of Tool used; Overall risk of bias judgement by study (e.g. Cochrane RoB1) or by outcome (e.g. Cochrane RoB2) [name all meta-review outcomes assessed where applicable] |
|  | Quality/Certainty of the evidence assessment | Yes/No from Methods /Results; Name of Tool used; Overall GRADE judgement by outcome [name all meta-review outcomes assessed where applicable] |
| Results | *Results - relative effect for comparison (95% CI), number of studies, p-value, I^2^ statistic, fixed /random effects model* | |
|  | Protocol outcome 1 | Name of meta-review protocol outcome; Name of review outcome (with units); Result |
|  | Protocol outcome 2 | Name of meta-review protocol outcome; Name of review outcome (with units); Result |
|  | Protocol outcome 3 | Name of meta-review protocol outcome; Name of review outcome (with units); Result |
|  | Protocol outcome 4 | Continued as above |
|  | Subgroup analysis | n=xx subgroups; Title of each subgroup analysis(s); Result of each subgroup analysis |
|  | Sensitivity analysis | n=xx sensitivity analyses; Title of each sensitivity analysis(s); Result of each sensitivity analysis |
|  | Funnel plot analysis | n=xx funnel plots; Title of each funnel plot(s); Result of each funnel plot |
| Full text | Planned meta-analysis but not done | Yes/No, Reason for not doing meta-analysis if Yes? |
|  | Outcomes where meta-analysis planned but not done | List outcomes where meta-analysis planned but not done or Not applicable |
|  | Meta-review protocol outcomes not reported by the review | List meta-review protocol outcomes not reported or Not applicable |
|  | Review outcomes not in meta-review protocol | List review-level outcomes reported but not in meta-review protocol or Not applicable |
|  | Funding | Yes/No, From where if Yes? |
|  | Additional notes | Any other key information not captured by data extraction fields |
|  | | |
| Full text | Classification as Prevention or Treatment or Management | |
|  | Definite Prevention | Yes/No 'Prevention' as reported by review authors in general text AND review authors do not report selection criteria for patients with ICU delirium AND all relevant included studies are described as prevention not treatment or management |
|  | Definite Treatment or Management | Yes/No 'Treatment or Management' as reported by review authors in general text AND review authors include delirium diagnosis in selection criteria AND all relevant included studies are described as treatment or management not prevention |
|  | Prevention/Treatment/ Management Status Unclear | Yes/No Yes/No Two or less of the above |
