# Supplementary material for: A systematic meta-review of interventions to prevent and manage delirium in the Intensive Care Unit: Part 1 – Pharmacological interventions
Source: Crit Care. 2025 Dec 30;29:540. doi: 10.1186/s13054-025-05615-0 (PMC12751364; doi:10.1186/s13054-025-05615-0)
Supplement: Supplementary file 3 — Additional file 2b: Exclusion reasons and references. [file 13054_2025_5615_MOESM3_ESM.docx]

**Additional file 2b: Exclusion reasons and references**

| **Exclusion reasons** | **Reviews (n = 41)** |
| --- | --- |
| Not ICU or <80% of included studies in ICU (n=15) | Chen 2016; Chen 2020; Dang 2023; Friedman 2014; Haque 2019; Khaing 2021; Kim 2020; Lin 2021; Liu 2022; Mu 2015; Pieri 2020; Shen 2018; Singh 2022; Zhang 2013; Zhu 2020 |
| No separate analysis for RCTs (n=9) | Bledowski 2012; Campbell 2019; Elefritz 2016; Huang 2021; Lin 2012; Smithburger 2019; Wang 2012; Zaal 2012; Zhou 2021 |
| POD population not reported as ICU (n=4) | Duan 2018; Poon 2023; Tao 2018; Xiong 2021 |
| Not a SR of RCTs (n=2) | Barr 2013; Reardon 2013 |
| Mixed adult/child population (≥12 years) with no separate analysis (n=1) | Zhang 2017 |
| Insufficient information in abstract or full text not retrieved (n=3) | Lin 2020; Mengjing 2023; Santos 2017 |
| Without meta-analysis, planned meta-analysis or reason for not doing meta-analysis (n=5) | Gerlach 2009; Lange 2021; Mo 2013; Roberts 2012; Rosenzweig 2015 |
| Overview with no additional eligible reviews identified (n=1) | Barbateskovic 2019 |
| Non-pharmacological review (n=1) | Bannon 2018 |

Abbreviations. SR: systematic review; RCT: randomised controlled trial; ICU: intensive care unit; POD: post-operative delirium; MA: meta-analysis

**Bannon 2018:**

Bannon L, McGaughey J, Verghis R, Clarke M, McAuley DF, Blackwood B. The effectiveness of non-pharmacological interventions in reducing the incidence and duration of delirium in critically ill patients: a systematic review and meta-analysis. Intensive Care Med. 2019;45(1):1-12.

**Barbateskovic 2019:**

Barbateskovic M, Krauss SR, Collet MO, et al. Pharmacological interventions for prevention and management of delirium in intensive care patients: a systematic overview of reviews and meta-analyses. BMJ Open 2019;9(2):e024562.

**Barr 2013:**

Barr J, Fraser GL, Puntillo K, Ely EW, Gélinas C, Dasta JF, Davidson JE, Devlin JW, Kress JP, Joffe AM, Coursin DB, Herr DL, Tung A, Robinson BR, Fontaine DK, Ramsay MA, Riker RR, Sessler CN, Pun B, Skrobik Y, Jaeschke R; American College of Critical Care Medicine. Clinical practice guidelines for the management of pain, agitation, and delirium in adult patients in the intensive care unit. Crit Care Med. 2013;41(1):263-306.

**Bledowski 2012:**

Bledowski J, Trutia A. A review of pharmacologic management and prevention strategies for delirium in the intensive care unit. Psychosomatics. 2012;53(3):203-11

**Campbell 2019:**

Campbell AM, Axon DR, Martin JR, Slack MK, Mollon L, Lee JK. Melatonin for the prevention of postoperative delirium in older adults: a systematic review and meta-analysis. BMC geriatrics. 2019;19:1-0.

**Chen 2016:**

Chen S, Shi L, Liang F, Xu L, Desislava D, Wu Q, Zhang J. Exogenous Melatonin for Delirium Prevention: a Meta-analysis of Randomized Controlled Trials. Mol Neurobiol. 2016;53(6):4046-4053.

**Chen 2020:**

Chen Z, Chen R, Zheng D, Su Y, Wen S, Guo H, Ye Z, Deng Y, Liu G, Zuo L, Wei X, Hou Y.  Efficacy and safety of haloperidol for delirium prevention in adult patients: An updated meta-analysis with trial sequential analysis of randomized controlled trials. J Clin Anesth. 2020;61:109623.

**Dang 2023:**

Dang V, Mansukhani MP, Wang Z, Kinzelman Vesely E, Kolla BP. Prophylactic Use of Ramelteon for Delirium in Hospitalized Patients: A Systematic Review and Meta-Analyses. J Acad Consult Liaison Psychiatry. 2023;64(1):65-72.

**Duan 2018:**

Duan X, Coburn M, Rossaint R, Sanders RD, Waesberghe JV, Kowark A. Efficacy of perioperative dexmedetomidine on postoperative delirium: systematic review and meta-analysis with trial sequential analysis of randomised controlled trials. Br J Anaesth. 2018;121(2):384-397.

**Elefritz 2016:**

Elefritz JL, Murphy CV, Papadimos TJ, Lyaker MR. Methadone analgesia in the critically ill. J Crit Care. 2016;34:84-8.

**Friedman 2014:**

Friedman JI, Soleimani L, McGonigle DP, Egol C, Silverstein JH. Pharmacological treatments of non-substance-withdrawal delirium: a systematic review of prospective trials. Am J Psychiatry. 2014;171(2):151-9.

**Gerlach 2009:**

Gerlach AT, Murphy CV, Dasta JF. An updated focused review of dexmedetomidine in adults. Ann Pharmacother. 2009;43(12):2064-74.

**Haque 2019:**

Haque N, Naqvi RM, Dasgupta M. Efficacy of Ondansetron in the Prevention or Treatment of Post-operative Delirium - a Systematic Review. Can Geriatr J. 2019;22(1):1-6.

**Huang 2021:**

Huang X, Lin D, Sun Y, Wu A, Wei C. Effect of Dexmedetomidine on Postoperative Sleep Quality: A Systematic Review. Drug Des Devel Ther. 2021;15:2161-2170.

**Khaing 2021:**

Khaing K, Nair BR. Melatonin for delirium prevention in hospitalized patients: A systematic review and meta-analysis. J Psychiatr Res. 2021;133:181–190.

**Kim 2020:**

Kim MS, Rhim HC, Park A, Kim H, Han KM, Patkar AA, Pae CU, Han C. Comparative efficacy and acceptability of pharmacological interventions for the treatment and prevention of delirium: A systematic review and network meta-analysis. J Psychiatr Res. 2020;125:164-176.

**Lange 2021:**

Lange S, Mędrzycka-Dąbrowska W, Friganovic A, Oomen B, Krupa S. Delirium in Critical Illness Patients and the Potential Role of Thiamine Therapy in Prevention and Treatment: Findings from a Scoping Review with Implications for Evidence-Based Practice. Int J Environ Res Public Health. 2021;18(16):8809.

**Lin 2021:**

Lin C, Tu H, Jie Z, Zhou X, Li C. Effect of Dexmedetomidine on Delirium in Elderly Surgical Patients: A Meta-analysis of Randomized Controlled Trials. Ann Pharmacother. 2021;55(5):624-636.

**Lin 2020:**

Lin P, Zhang J, Shi F, Liang Z-A. Can haloperidol prophylaxis reduce the incidence of delirium in critically ill patients in intensive care units? A systematic review and meta-analysis. Heart Lung. 2020;49:265–272.

**Lin 2012:**

Lin YY, He B, Chen J, et al. Can dexmedetomidine be a safe and efficacious sedative agent in post-cardiac surgery patients? a metaanalysis. Crit Care 2012;16:R169.

**Liu 2022:**

Liu X, Hu Q, Chen Q, Jia J, Liao YH, Feng J. Effect of dexmedetomidine for prevention of acute kidney injury after cardiac surgery: an updated systematic review and meta-analysis. Ren Fail. 2022;44(1):1150-1159.

**Mengjing 2023:**

Mengjing HAN, Wenjia T, Hongqian W, Xuewen F, Pin S, Yingping FU. Effect of early comfort using analgesia, minimal sedatives and maximal humane care strategy on delirium in ICU patients with mechanical ventilation: a meta-analysis. Chinese Evidence-based Nursing. 2023 9(5):761-768.

**Mo 2013:**

Mo Y, Zimmermann AE. Role of Dexmedetomidine for the Prevention and Treatment of Delirium in Intensive Care Unit Patients. Annals of Pharmacotherapy [Internet]. 2013;47(6):869–76.

**Mu 2015:**

Mu JL, Lee A, Joynt GM. Pharmacologic agents for the prevention and treatment of delirium in patients undergoing cardiac surgery: systematic review and metaanalysis. Crit Care Med. 2015;43(1):194-204.

**Pieri 2020:**

Pieri M, De Simone A, Rose S, De Domenico P, Lembo R, Denaro G, et al. Trials Focusing on Prevention and Treatment of Delirium After Cardiac Surgery: A systematic Review of Randomized Evidence. Journal of Cardiothoracic and Vascular Anesthesia [Internet]. 2020;34(6):1641–54.

**Poon 2023:**

Poon WH, Ling RR, Yang IX, Luo H, Kofidis T, MacLaren G, Tham C, Teoh KLK, Ramanathan K. Dexmedetomidine for adult cardiac surgery: a systematic review, meta-analysis and trial sequential analysis. Anaesthesia. 2023;78(3):371-380.

**Reardon 2013:**

Reardon DP, Anger KE, Adams CD, Szumita PM. Role of dexmedetomidine in adults in the intensive care unit: An update. American Journal of Health-System Pharmacy [Internet]. 2013;70(9):767–77.

**Roberts 2012:**

Roberts DJ, Haroon B, Hall RI. Sedation for Critically Ill or Injured Adults in the Intensive Care Unit. Drugs [Internet]. 2012;72(14):1881–916.

**Rosenzweig 2015:**

Rosenzweig AB, Sittambalam CD. A new approach to the prevention and treatment of delirium in elderly patients in the intensive care unit. J Community Hosp Intern Med Perspect. 2015;5(4):27950.

**Santos 2017:**

Santos E, Cardoso D, Neves H, Cunha M, Rodrigues M, Apóstolo J. Effectiveness of haloperidol prophylaxis in critically ill patients with a high risk of delirium: a systematic review. JBI Database System Rev Implement Rep. 2017;15(5):1440-1472.

**Shen 2018:**

Shen YZ, Peng K, Zhang J, Meng XW, Ji FH. Effects of Haloperidol on Delirium in Adult Patients: A Systematic Review and Meta-Analysis. Med Princ Pract. 2018;27(3):250-259.

**Singh 2022:**

Singh A, Broad J, Brenna CTA, Kaustov L, Choi S. The Effects of Dexmedetomidine on Perioperative Neurocognitive Outcomes After Noncardiac Surgery: A Systematic Review and Meta-Analysis of Randomized Controlled Trials. Ann Surg Open. 2022;3(1):e130.

**Smithburger 2019:**

Smithburger PL, Patel MK. Pharmacologic considerations surrounding sedation, delirium, and sleep in critically ill adults: a narrative review. Journal of pharmacy practice. 2019;32(3):271-91.

**Tao 2018:**

Tao R, Wang XW, Pang LJ, Cheng J, Wang YM, Gao GQ, Liu Y, Wang C. Pharmacologic prevention of postoperative delirium after on-pump cardiac surgery: A meta-analysis of randomized trials. Medicine (Baltimore). 2018;97(43):e12771.

**Wang 2012:**

Wang EH, Mabasa VH, Loh GW, Ensom MH. Haloperidol dosing strategies in the treatment of delirium in the critically ill. Neurocrit Care. 2012;16(1):170-83.

**Xiong 2021:**

Xiong X, Chen D, Shi J. Is Perioperative Dexmedetomidine Associated With a Reduced Risk of Perioperative Neurocognitive Disorders Following Cardiac Surgery? A Systematic Review and Meta-Analysis With Trial Sequential Analysis of Randomized Controlled Trials. Front Med (Lausanne). 2021;8:645975.

**Zaal 2012:**

Zaal IJ, Slooter AJC. Delirium in Critically Ill Patients. Drugs [Internet]. 2012;72(11):1457–71.

**Zhang 2013:**

Zhang H, Lu Y, Liu M, et al. Strategies for prevention of postoperative delirium: a systematic review and meta-analysis of randomized trials. Crit Care 2013;17:R47.

**Zhang 2017:**

Zhang Z, Chen K, Ni H, Zhang X, Fan H. Sedation of mechanically ventilated adults in intensive care unit: a network meta-analysis. Sci Rep. 2017;7:44979.

**Zhou 2021:**

Zhou W-J, Liu M, Fan X-P (2021) Differences in efficacy and safety of midazolam vs. dexmedetomidine in critically ill patients: A meta-analysis of randomized controlled trial. Exp Ther Med 21:156.

**Zhu 2020:**

Zhu Y, Jiang Z, Huang H, et al (2020) Assessment of Melatonergics in Prevention of Delirium: A Systematic Review and Meta-Analysis. Front Neurol 11:198.
