# Supplementary material for: A systematic meta-review of interventions to prevent and manage delirium in the Intensive Care Unit: Part 1 – Pharmacological interventions
Source: Crit Care. 2025 Dec 30;29:540. doi: 10.1186/s13054-025-05615-0 (PMC12751364; doi:10.1186/s13054-025-05615-0)
Supplement: Supplementary file 4 — Additional file 3a: Included review characteristics - population. [file 13054_2025_5615_MOESM4_ESM.docx]

**Additional file 3a: Included review characteristics - population**

| **Review** | **Review population^1^** | **Included countries (multicentre or single centre RCTs)^2^** | **Age (years)^3^** | **Sex or Gender^3^** | **Ethnicity^3^** |
| --- | --- | --- | --- | --- | --- |
| Afzal 2023 | Critically ill | China; India; Netherlands; United States | Study-level: 'mean age of patients…ranged from 53.7 to 74.2 years' | '…the majority of participants were male' | - |
| Aiello 2023 | Critically ill | - | Study-level: mean age ranged from 34.62 years (melatonin group) to 71.6 years (placebo group) or 78.3 years (ramelteon group, RCT in sensitivity analysis only) | Not reported | - |
| Aitken 2021 | Critically ill, mechanically ventilated | Australia; Brazil; Denmark; New Zealand; Norway; Sweden; Switzerland; United States | - | - | - |
| Al-Qadheeb 2014 | Critically ill | - | ‘The average patient age was older than 50 years’ | The proportion of males varied from 41% (non-pharmacological intervention) or 45% (pharmacological control) to 90% (pharmacological intervention) | - |
| Barbateskovic 2020 | Critically ill | - | 'Mean age of participants ranged from 31 years to 71 years' | '…proportion of men ranged between 54% and 91%' | - |
| Burry 2014 | Critically ill, mechanically ventilated | Australia; Brazil; Canada; Greece; Turkey; United States | Study-level: average ≥44.76 years but mixed reporting of mean or median values or not reported | Study-level: 27.1-59% female or not reported | - |
| Burry 2019 | Critically ill | Australia; Canada; Egypt; Netherlands; New Zealand; Turkey; United Kingdom; United States | Study-level: mean age 32 ± 5 years to elderly (aged ≥65) | Study-level: 43-94% male | - |
| Burry 2021 | Critically ill | Primarily North America, Europe and Asia | 'The mean or median age at randomization ranged from 34.6 to 77.4 years, and 56 (70%) of trials reported a mean or median age of 60 or greater' | - | - |
| Chen 2015 | Critically ill, mechanically ventilated | Australia; Belgium; China; Estonia; Finland; France; Germany; Netherlands; New Zealand; Norway; Russian Federation; Switzerland; United Kingdom; United States | Study-level: median 58.77 years (midazolam group) to median 68 years (standard care group) | - | - |
| Constantin 2016 | Critically ill | Australia; Argentina, Belgium, Brazil, China; Egypt; Finland; Germany; Japan; Netherlands; New Zealand; Russia; Switzerland; Turkey; United Kingdom; United States | - | - | - |
| Cruickshank 2016 | Critically ill, mechanically ventilated | Argentina; Australia; Canada; Brazil; Egypt; Europe; Finland; India; New Zealand; Russia; Switzerland; Turkey; United Kingdom; United States | '...With the exception of one trial that focused exclusively on young pregnant women (mean age 25.1 years in the dexmedetomidine group and 26.8 years in the control intervention group), the 11 remaining trials mean age ranged from 43 to 65 years for dexmedetomidine and from 40 to 67 years for the comparator interventions. The median age was reported in six trials and ranged from 49 to 65 years for dexmedetomidine and from 46 to 67 years for the comparator interventions’ | ‘Study populations tended to involve more men than women, with the exception of one trial that involved only pregnant women' | - |
| Cuninghame 2023 | Critically ill | - | For RCTs, the median reported age was 52-65 years | 16-55% female in RCTs | - |
| Dong 2020 | Critically ill, mechanically ventilated | - | Study-level: median age (based on reporting in 3 RCTs) 59-65 years; mean age (based on reporting in 4 RCTs) 61.5-69 years ± 7.3-17 | Study-level: intervention group males 41.37-78.4%; Control group males 35-86.3% | - |
| Duan 2023 | Critically ill | - | Study-level: overall RCT mean age ranged from 49.9 ± 19.0 to 71.6 years ± 6.6 | >50% male | - |
| Fan 2017 | Critically ill, post-surgery | - | ‘…older on average (64±23 yr old)’ | - | - |
| Flukiger 2018 | Critically ill | Australia; Canada; China; Finland; Europe; India; Japan; New Zealand; Russia; South Korea; Switzerland Taiwan; Turkey; United States | Study-level: mean age ranged from 34.6 to 75 years | Study-level: 37.5-85% male and not reported for one trial | - |
| Fraser 2013 | Critically ill, mechanically ventilated | - | 'On average, patients were older (mean age = 59 yr)’ | - | - |
| Herling 2018 | Critically ill | Australia; Belgium; Brazil; Canada; Denmark; Egypt; Netherlands; New Zealand; Republic of Korea; United Kingdom; United States | '…mean 48 to 70 years’ | Study-level: ≥50 to 74% of intervention or comparator groups male | - |
| Heybati 2022 | Critically ill, mechanically ventilated | Canada; China; Egypt; Finland; India; Italy; Japan; Russia; Switzerland; Turkey; United Kingdom United States | ‘…mean age ranged from 34.56 to 83.10 yr’ | ‘32.08% of the patients were female’ | - |
| Huang 2023 | Critically ill | - | Study-level: 51-83.5 years | Study-level: 41.1 to 71.6% of intervention or comparator groups male | - |
| Leigh 2019 | Critically ill | Greece, Turkey | Study-level: mean age 58.91 ± 10.49 to 70.90 ± 9.90 | - | - |
| Lewis 2021 | Critically ill, mechanically ventilated | China; Egypt; France; Poland; Russia; Saudia Arabia; Spain Turkey; Ukraine; United States | Mean age 61.5 ± 6.8 years | 'On average, 36.1% of participants were women' | - |
| Lewis 2022 | Critically ill, mechanically ventilated | Australia; Brazil; Canada; China; Egypt; Europe; Finland; Germany; Greece; India; Iran; Italy; Japan; Korea; Malaysia; New Zealand; Russia; South Korea; Switzerland; Taiwan, Turkey; United Kingdom; United States; Not reported | 'overall mean age of 58.8 ± 10.2 years’ | ‘Approximately 39% of participants were female' | - |
| Liu 2021 | Critically ill | America; Australia; China; New Zealand; Pakistan; Russia; Saudia Arabia; Turkey | - | - | - |
| Liu 2023 | Critically ill | Canada; China; Iran | - | - | - |
| Lonergan 2009 | Hospitalised including mechanically ventilated | - | Study-level: 'average age in the dexmedetomidine group was 60 (49 to 65) and in the lorazepam group, 49 (45 to 57)' | Study-level: '30 (58%) men in the dexmedetomidine group and 23 (45%) in the lorazepam group' | - |
| Long 2020 | Critically ill, mechanically ventilated | - | Overall range 47 to 69 years | - | - |
| Luo 2019 | Critically ill | United Kingdom; United States | Study-level: 52.5 years; 62 years | Study-level: 42% female; 50% female | - |
| Marra 2021 | Critically ill | - | Study-level: mean age 49-74 years or not reported | - | - |
| Mukundarajan 2023 | Critically ill | - | Study-level: average ≥55 years but mixed reporting of mean, median and range values or not reported | - | - |
| Nassar 2016 | Critically ill | Brazil; Canada; Denmark; Greece; Turkey; United States | - | - | - |
| Nelson 2015 | Critically ill | - | Average ≥55 years but mixed reporting of mean and median values | Study-level: 51.5% male; 63.6% male; 75.3% male | - |
| Ng 2019 | Critically ill | Australia; Canada; China; Egypt; Europe; Finland; Germany; India; Japan; Korea; New Zealand; Turkey; Ukraine; United Kingdom; United States | - | - | - |
| Pasin 2014 | Critically ill | - | - | - | - |
| Peng 2017 | Critically ill, mechanically ventilated | - | - | - | - |
| Pereira 2020 | Critically ill | Canada; China; Taiwan; United States | >60 years | - | - |
| Porhomayon 2015 | Critically ill | - | - | - | - |
| Qi 2021 | Critically ill, mechanically ventilated | Australia; China; Iran | Study-level: mean age 38.3 ± 6.4 to 66.8 ± 17.7 | Study-level: 57.8-81.4% male or not reported | - |
| Sattar 2023 | Critically ill, post-surgery | China; Egypt; India; Russia; Turkey; United States | Study-level: mean 33.6 to 72.7 years by study intervention or comparator group or not reported | Study-level: 30-87.9% male by intervention or comparator group or not reported | - |
| Sedhai 2021 | Critically ill | - | Average ≥53 years but mixed reporting of mean and median values | Majority male except for one study control group | - |
| Serafim 2015 | Critically ill | - | - | - | - |
| Tan 2010 | Critically ill | Australia; Egypt; Finland; Germany; Japan; Switzerland; Turkey; United Kingdom; United States | - | - | - |
| Tran 2018 | Critically ill, mechanically ventilated | China; India; United States | Study-level: average ≥35.6 years ± 10.7 to 54.1 years ±17.8 or not reported | Study-level: 43-83% male or not reported | - |
| Wang 2021 | Critically ill | Australia; Argentina; Brazil; Canada; China; Egypt; India; Ireland; Italy; Japan; Korea; Malaysia; Netherlands; New Zealand; Saudia Arabia; Switzerland, Taiwan; United Kingdom; United States | ‘mean/median ages of included patients ranged from 41.4 years to 76 years’ | Study-level: 43.5-84.2% male | - |
| Wang 2019a | Critically ill, mechanically ventilated | - | Study-level: mean age 27 years ± 7 to 68 years ± 48.1) | - | - |
| Wang 2019b | Critically ill, mechanically ventilated | Brazil; Denmark; Egypt; Europe; Germany | - | - | - |
| Wu 2022 | Critically ill | - | Range 26-81 years but with mixed reporting of range and average values | - | - |
| Xia 2013 | Critically ill | - | - | - | - |
| Xing 2018 | Critically ill, mechanically ventilated | Canada; Switzerland; Turkey; United States | Study-level: mean age ≥55 to 72.7 years or not reported | Majority male across intervention groups except in one study | - |
| Yan 2022 | Critically ill | Australia; Brazil; China; Egypt; Germany; India; Iran; Japan; United States | Study-level: mean ≥37.3 to 71.6 years | Study-level: 49.6-77.8% male | - |
| Yang 2021 | Critically ill | America; Austria; China; Denmark; Germany; Greece; Italy; Korea; Oman; multiple countries | - | - | - |
| Yiewong 2023 | Critically ill, post-surgery (and mechanically ventilated) | Australia; China; Ireland; Italy; Malaysia; New Zealand; Saudi Arabia; Switzerland; United Kingdom | - | - | - |
| Zayed 2019 | Critically ill | - | 'mean age 66.59 ± 13.18' | '57.8% were males' (ranging from 52-63.3%) | - |
| Zhang 2019 | Critically ill | Australia; China; India; Iran; Japan; United Kingdom; United States | Study-level: mean age 36.9 years ± 10.3 to 78.3 years ± 6.8 | Majority male across intervention and control groups except in two studies; not reported in one study | - |
| Zhang 2022 | Critically ill | - | 'The mean participant age ranged from 43 to 75 years' (mean or median reported) | Across all included studies, the proportion of males was '58.9% of the dexmedetomidine group and 56.8% of the control group' | - |
| Zitikyte 2023 | Critically ill, trauma | Egypt; Iran; Spain; United States | Study-level: mean age 32 to 53.2 years | Study-level: 70-88% male | - |

Footnotes:

^1^ This included trials of patients identified as having ICU delirium, being at high risk of developing delirium, and several trials identified as targeting patients with subsyndromal delirium. ^2^ RCT: randomised controlled trial. ^3^ Fields extracted for included RCTs or at the review level where available.
