# Supplementary material for: A systematic meta-review of interventions to prevent and manage delirium in the Intensive Care Unit: Part 1 – Pharmacological interventions
Source: Crit Care. 2025 Dec 30;29:540. doi: 10.1186/s13054-025-05615-0 (PMC12751364; doi:10.1186/s13054-025-05615-0)
Supplement: Supplementary file 5 — Additional file 3b: Included review characteristics - interventions, comparators and outcomes. [file 13054_2025_5615_MOESM5_ESM.docx]

**Additional file 3b: Included review characteristics – interventions, comparators and outcomes**

| **Review** (RCTs)^1^ | **Review Intervention vs Comparison** | **Meta-analysed Core Outcomes^2^** | **Other Composite Delirium Outcomes^3^** |
| --- | --- | --- | --- |
| Afzal 2023 (7) | Typical antipsychotic and atypical antipsychotic vs each other or placebo | Duration of delirium (days); ICU length of stay; Mortality |  |
| Aiello 2023 (9) | Melatonin and/or ramelteon vs placebo or no other treatment | Delirium incidence; ICU length of stay; Mortality |  |
| Aitken 2021 (8) | ‘Lighter’ sedation vs ‘Deeper sedation’ | Delirium (%); ICU length of stay; Hospital length of stay; Mortality |  |
| Al-Qadheeb 2014 (17; 13 drug interventions) | Any ‘pharmacological, non-pharmacological or mixed pharmacological/ non-pharmacological strategy hypothesized to decrease delirium burden’ vs standard care or placebo | Duration of delirium (days); Mortality |  |
| Barbateskovic 2020 (8) | Haloperidol vs any intervention | Mortality; Delirium severity |  |
| Burry 2014 (9) | Daily sedation interruption vs no daily sedation interruption (usual care or protocolized sedation strategies) | ICU length of stay; Hospital length of stay; Mortality; Quality of life. Other: adverse events including new-onset delirium |  |
| Burry 2019 (14) | Any drug (including α2-adrenoceptor agonists, antidepressants, antipsychotics (either typical or atypical agents), benzodiazepines, cholinesterase inhibitors, N-methyl-D-aspartate receptor antagonist, melatonin and melatonin agonists, opioids, propofol, serotonin receptor antagonists and statins) vs another active drug treatment, a placebo, or a non-drug intervention) | Duration of delirium (no units, log ratio of means); ICU length of stay; Hospital length of stay; Mortality | Delirium-free and coma-free days |
| Burry 2021 (80; 54 drug or sedation strategy studies) | Any drug, non-drug or multi-component intervention or sedation strategy | Delirium occurrence (incidence or prevalence); Delirium duration; ICU length of stay; hospital length of stay; Mortality. Other: adverse events, arrhythmia | Delirium-free and coma-free days |
| Chen 2015 (6 studies; 7 RCTs) | Alpha-2 adrenoceptor agonists (dexmedetomidine) for sedation of more than 24 hours vs traditional sedatives (including benzodiazepine derivatives (midazolam or lorazepam), propofol, combinations of sedatives and other sedatives e.g. ‘standard care’) | Risk of delirium; ICU length of stay; Mortality. Other: adverse events, bradycardia, hypotension |  |
| Constantin 2016 (15 studies; 16 RCTs) | Dexmedetomidine vs another sedative agent, with or without opioid association | Delirium incidence; ICU length of stay; Mortality. Other: adverse events, bradycardia, hypotension |  |
| Cruickshank 2016 (18) | Alpha-2 adrenoceptor agonists for sedation (dexmedetomidine vs clonidine or combined) vs propofol or benzodiazepines (such as midazolam, lorazepam, and diazepam) | Delirium incidence (timing not reported); ICU length of stay; Mortality. Other: adverse events, bradycardia, tachycardia, hypertension, hypotension, self-extubation |  |
| Cuninghame 2023  (13 studies; 8 RCTs) | Inhaled anaesthetics (sevoflurane, isoflurane, or desflurane) for sedation. Volatile anaesthetics vs intravenous anaesthesia (e.g., propofol, midazolam) | Delirium incidence |  |
| Dong 2020 (7) | Dexmedetomidine vs midazolam/propofol, lorazepam, midazolam or ‘unclear’ | Delirium incidence; ICU length of stay; Mortality. Other: adverse events |  |
| Duan 2023 (6 studies; 4 RCTs) | Melatonin vs placebo | Mortality |  |
| Fan 2017 (16) | Dexmedetomidine vs propofol or placebo | Delirium prevalence; ICU length of stay; Hospital length of stay. Other: adverse events, hypotension, bradycardia, tachycardia |  |
| Flukiger 2018 (28; incidence (25 RCTs); treatment (3 RCTs)) | Dexmedetomidine vs any comparator. (Comparator divided as placebo, standard sedatives (including propofol, midazolam, and lorazepam), and opioids (including morphine and remifentanil)) | Delirium incidence; for treatment: Delirium resolution; ICU length of stay. Other: adverse events including mortality |  |
| Fraser 2013 (6) | Benzodiazepine vs non-benzodiazepine-based sedation (dexmedetomidine vs midazolam; midazolam vs propofol; lorazepam vs propofol; dexmedetomidine vs lorazepam) | Delirium prevalence (during ICU stay); ICU length of stay; Mortality |  |
| Herling 2018 (12; 8 drug/sedation strategy RCTs) | ‘any non-pharmacological intervention, single or multicomponent, including cognitive training i.e. digit span forwards/backwards, letter-number sequences, sudokus, early mobilization, modification of ICU environment through lighting or ear plugs, physical therapy or pharmacological interventions, or both’ vs standard care, placebo, or both | Rate of ICU delirium (in 28 days); ICU length of stay; Mortality | Delirium-free and coma-free days |
| Heybati 2022 (41) | Dexmedetomidine vs propofol | ICU delirium incidence; Duration of delirium; ICU length of stay; Mortality. Other: adverse events, bradycardia, tachycardia, hypotension, hypertension |  |
| Huang 2023 (9) | Haloperidol vs placebo or ‘no any type of intervention’ | ICU length of stay; Hospital length of stay; Mortality (with/without Delirium). Other: adverse events |  |
| Leigh 2019 (3) | Any pharmacological intervention (including ‘atypical and typical antipsychotics, such as, but not limited to, haloperidol, quetiapine and olanzapine; benzodiazepines, such as, but not limited to, diazepam and midazolam; sedatives, such as, but not limited to, propofol; and alpha-agonists, such as, but not limited to, dexmedetomidine and clonidine.’ Drug comparators including any two pharmacological interventions from the previously mentioned classes of drugs) | - |  |
| Lewis 2021 (12) | Dexmedetomidine vs another sedative (including, but not limited to, propofol, antipsychotics, opioids, or benzodiazepines) or placebo | Risk of delirium (during ICU stay); ICU length of stay; Mortality. Other: adverse events, pneumonia, bradycardia, hypotension |  |
| Lewis 2022 (77) | Dexmedetomidine vs another sedative, usual care, or placebo | Risk of delirium (timing not reported); ICU length of stay; Hospital length of stay; Mortality. Other: adverse events, any, bradycardia, hypotension, hypertension, self-extubation, agitation |  |
| Liu 2021 (15 studies; 10 RCTs | Dexmedetomidine vs placebo or any other drug for the treatment of delirium | Delirium point-prevalence after treatment (last time point); Time to resolution of delirium (hours); ICU length of stay. Other: adverse events, hypotension, bradycardia |  |
| Liu 2023 (9 studies; 4 RCTs) | Olanzapine vs ‘other interventions, including routine care (no intervention), nonpharmaceutical interventions and pharmaceutical interventions’ | Delirium duration meta-analysed but the index comparator was a traditional Chinese medicine (Taohe Chengqi Decoction) in 1 of 2 included RCTs. Other: overall adverse events included Taohe Chengqi Decoction as the comparator in 1 of 3 included RCTs |  |
| Lonergan 2009 (1) | ‘benzodiazepines, of any dosage, compared with placebo or another drug’ short-acting benzodiazepine, lorazepam vs selective  α2-adrenoceptor agonist, dexmedetomidine | - | Duration of coma- and delirium-free days; Prevalence of delirium or coma |
| Long 2020 (18 studies; 10 RCTs) | Lighter sedation vs Deeper sedation | Delirium incidence; Mortality. Other: adverse events, agitation-related adverse events |  |
| Luo 2019 (9 studies; 2 RCTs) | Any member of the statin family within the licensed dose range vs placebo | Delirium incidence (daily delirium status in intensive care up to 28 days) |  |
| Marra 2021  (7 or 8; 7 with study information) | Haloperidol vs placebo | Delirium incidence; Delirium duration; ICU length of stay; Hospital length of stay; Mortality. Other: adverse events, extrapyramidal effects | Delirium-free and coma-free days |
| Mukundarajan 2023 (12) | Prophylactic melatonin or ramelteon (melatonergics) vs placebo or ‘standard therapy’ | Delirium incidence; ICU length of stay; Hospital length of stay; Mortality; Delirium severity |  |
| Nassar 2016 (7) | Daily sedation interruption vs Sedation protocols | Delirium occurrence/ Prevalence of delirium; Mortality |  |
| Nelson 2015 (3) | Dexmedetomidine vs other non-dexmedetomidine sedation strategies | - |  |
| Ng 2019 (25) | Dexmedetomidine vs placebo | ICU delirium incidence; ICU length of stay; Hospital length of stay; Mortality. Other: adverse events, bradycardia, hypotension |  |
| Pasin 2014 (13 publications, 14 RCTs) | Dexmedetomidine vs any comparator (with subgroup analyses for dexmedetomidine vs midazolam and dexmedetomidine vs propofol) | - | Delirium incidence/ rate as a composite with agitation and/or confusion |
| Peng 2017  (6 controlled trials -interpreted as RCTs as ‘adequate sequence generation’) | Dexmedetomidine vs midazolam | Post-operative delirium risk. Unclear if post-operative delirium (POD) as distinct from ICU delirium |  |
| Pereira 2020 (8 studies; 6 RCTs) | Dexmedetomidine vs propofol sedation | Delirium incidence/ occurrence; ICU length of stay; Hospital length of stay. Other: adverse events, hypotension |  |
| Porhomayon 2015  (11 studies; 6 RCTs) | Heavy vs Light sedation dosing strategies | Delirium |  |
| Qi 2021 (14 studies; 8 RCTs) | Nurse-led sedation protocols vs Physician-led usual care | Delirium incidence; ICU length of stay; Mortality |  |
| Sattar 2023 (14) | Dexmedetomidine vs propofol | Risk of delirium/ Number of patients diagnosed with delirium; ICU length of stay; Hospital length of hospital stay. Other: adverse events, bradycardia, atrial fibrillation, hypotension |  |
| Sedhai 2021 (19 studies; 8 RCTs) | Thiamine and standard of care vs placebo and/or standard of care | Delirium incidence; ICU length of stay; Hospital length of stay; Mortality rate |  |
| Serafim 2015  (21 studies; 7 RCTs of ‘treatment’ with one study considered prevention and treatment) | Mixed index drugs. Comparator not specified at review level (4 RCTs reported to involve a placebo) | ICU length of stay. No separate meta-analysis for RCTs/non-RCTs on prevention |  |
| Tan 2010 (24) | Dexmedetomidine vs placebo or another sedative agent (e.g., propofol or benzodiazepines) | Risk of delirium; ICU length of stay; Hospital length of stay (incompletely reported); Mortality. Other: adverse events, bradycardia, hypotension, atrial fibrillation, nausea and vomiting, myocardial infarction, hyperglycemia |  |
| Tran 2018 (17 studies; 6 RCTs) | Alpha-2 adrenoceptor agonists for ‘non-procedural sedation’ vs any analgo-sedative regimen including but  not limited to propofol, benzodiazepines, opioids, or ketamine | - |  |
| Wang 2021 (39 publications; 36 RCTs) | Sedation protocols with/without dexmedetomidine (10 studies used a placebo) | Risk of delirium; Duration of delirium (days); ICU length of stay; Hospital length of stay; Mortality. Other: adverse events, hypotension, bradycardia, hypoxaemia, hypotension |  |
| Wang 2019a (31) | Individual and combined sedative drugs | Number of patients with delirium; ICU length of stay; Mortality rate. Other: adverse events, hypotension |  |
| Wang 2019b (17 studies; 6 RCTs) | Analgesia-based sedation or analgosedation or no sedation. Comparisons included sedation strategies (e.g. none or intermittent vs daily interruption, protocolized vs non-protocolised) and analgesia-based vs hypnotic-based sedation | Delirium incidence/ rate; Mortality |  |
| Wu 2022 (5) | Dexmedetomidine intravenous infusion. Comparator incompletely reported at review level | Incidence of ‘restless delirium’; Duration of total delirium after medication; ICU length of stay. Other: adverse events (bradycardia and hypotension) |  |
| Xia 2013 (10) | Dexmedetomidine vs propofol for sedation | Risk of delirium; ICU length of stay; Mortality. Other: Adverse events, hypotension, bradycardia, hypertension |  |
| Xing 2018 (8) | Dexmedetomidine vs midazolam/ propofol | Delirium incidence; ICU length of stay |  |
| Yan 2022 (12) | Prophylactic melatonin or melatonin receptor agonists (ramelteon) vs placebo, standard of care or no intervention | Delirium prevalence; Duration of delirium; ICU length of stay; Hospital length of stay; Mortality |  |
| Yang 2021 (15) | Remifentanil vs other opioids | ICU length of stay; Hospital length of stay; Mortality. Other: adverse events, delirium, nausea/ vomiting, hypotension, dysrhythmia, side effects |  |
| Yiewong 2023 (5) | Early sedation with dexmedetomidine ‘including nocturnal low-dose intravenous dexmedetomidine infusion’ vs ‘medication other than dexmedetomidine for the promotion of sleep quality. The other medications included but were not confined to propofol, morphine, or a placebo’ | Delirium incidence |  |
| Zayed 2019 (6) | Haloperidol vs placebo | Delirium incidence; ICU length of stay; Mortality. Other: adverse events, serious adverse events, QTc prolongation, extrapyramidal symptoms | Delirium-free and coma-free days |
| Zhang 2019 (8; 7 ICU) | Exogenous melatonin and melatonin receptor agonists (7 studies in ICU were placebo-controlled) | Delirium prevalence; ICU length of stay; Mortality |  |
| Zhang 2022 (19) | Dexmedetomidine vs other sedatives | Delirium incidence; ICU length of stay; Mortality. Other: adverse events (total including tachycardia, bradycardia, and hypotension), arrhythmia, hypotension |  |
| Zitikyte 2023 (6) | Any pharmacological interventions | Delirium prevalence / occurrence (timing not reported); ICU length of stay; Hospital length of stay; Mortality |  |

Footnotes:

^1^ RCT: randomised controlled trial. ^2^ Meta-analysis from included review. ^3^ Composite delirium outcome not included in the Del-CorS core outcome set or meta-review mapping but extracted from the included review. Note: non-specific adverse event reporting was also sometimes found to include delirium - see ‘Meta-analysed Core Outcomes’.
