# Supplementary material for: A systematic meta-review of interventions to prevent and manage delirium in the Intensive Care Unit: Part 1 – Pharmacological interventions
Source: Crit Care. 2025 Dec 30;29:540. doi: 10.1186/s13054-025-05615-0 (PMC12751364; doi:10.1186/s13054-025-05615-0)
Supplement: Supplementary file 6 — Additional file 4: Pharmacological effect estimates for mapped ICU delirium outcomes. [file 13054_2025_5615_MOESM6_ESM.docx]

**Additional file 4: Pharmacological effect estimates for mapped ICU delirium outcomes**

| **Review** | **Drug / Drug class / Sedation strategy** | **Comparator** | **Delirium occurrence** (no units) | **Delirium duration** (days or no units) | **Delirium severity**  (no units) |
| --- | --- | --- | --- | --- | --- |
| **Alpha-2 adrenoceptor agonists / Dexmedetomidine** | | | | | |
| Burry 2021 | ‘Alpha-2 adrenoceptor agonists’ | Placebo | OR 0.43 (95% CrI 0.21, 0.85); direct evidence: 5 studies (1055 participants); moderate certainty  NMA effect estimate |  |  |
| Burry 2021 | Dexmedetomidine | Benzodiazepine | OR 0.21, (95% CrI 0.08, 0.51); direct evidence; 4 studies (473 participants); low certainty  NMA effect estimate |  |  |
| Ng 2019 | Dexmedetomidine | Placebo | OR 0.36 (95% CI 0.26, 0.51); p=0.0001; I^2^=0%; 8 studies (1425 participants); fixed effects model; high certainty |  |  |
| Heybati 2022^*^ | Dexmedetomidine | Propofol | RR 0.50 (95% CI 0.29, 0.87); p=0.019; I^2^=21%; 10 studies (801 participants); random effects model; high certainty |  |  |
| Heybati 2022^*^ | Dexmedetomidine | Propofol |  | MD -31.19 (95% CI -62.67, 0.30); p=not reported; I^2^=96%; 3 studies (363 participants); random/fixed effects model not reported; very low certainty |  |
| Lewis 2021 | Dexmedetomidine | Mixed (other sedation strategies) | RR, 0.34 (95% CI 0.22, 0.54); p<0.00001; I^2^=0%; 7 studies (537 participants); random effects model; moderate certainty |  |  |
| Lewis 2022 | Dexmedetomidine | Mixed (other sedatives) | RR 0.67 (95% CI 0.55, 0.81); p<0.0001; I^2^=64%; 33 studies (7958 participants); random effects model; moderate certainty^1^ |  |  |
| Zhang 2022 | Dexmedetomidine | Mixed (other sedation types) | RR 0.98 (95% CI 0.72, 1.33); p=0.88; I^2^=0%; 2 studies (264 participants); random effects model; low certainty |  |  |
| Wang 2021 | Dexmedetomidine | Mixed (non-dexmedetomidine) | RR 0.63 (95% CI 0.54, 0.75; p=0.0001; I^2^=65%; 33 studies (9478 participants); very low certainty^1^ | MD -0.74 d (95% CI -1.83, 0.36); p=0.19; I^2^=95%; 6 studies (1050 participants); very low certainty |  |
| **Antipsychotics** | | | | | |
| Burry 2021 | All | Placebo | OR 0.63 (95% CrI 0.36, 1.04); direct evidence: 8 studies (2776 participants); low certainty  NMA effect estimate |  |  |
| Burry 2019 | Typical | Placebo |  | RoM 0.96 (95% CrI 0.64, 1.36); direct evidence: 4 studies (608 participants); high certainty^2^  NMA effect estimate |  |
| Burry 2019 | Atypical | Placebo |  | RoM 0.80 (95% CrI 0.50, 1.11); direct evidence: 4 studies (500 participants); moderate certainty^3^  NMA effect estimate |  |
| Barbateskovic 2020 | Haloperidol | Mixed (chlorpromazine, lorazepam, risperidone or ondansetron) |  |  | SMD -0.15 (95% CI -0.61, 0.30); p=0.51; I^2^=27%; 3 studies, 4 comparisons (134 participants); random effects model; very low certainty^4^ |
| **Melatonergics** | | | | | |
| Mukundarajan 2023 | Melatonin / Ramelteon | Placebo / Standard | OR 0.63 (95% CI 0.30, 1.32); p=0.22; I^2^=71%; 7 Studies (1330 participants); random effects model; high certainty^5^ |  | MD 0.22 (95% CI -1.36, 1.81); p=0.78; I^2^=89%; 2 studies (225 participants); random effects model; high certainty^6^ |
| Burry 2021 | Melatonin / MRA | Placebo | OR 0.66 (95% CrI 0.19, 2.50); direct evidence: 2 studies (225 participants); low certainty  NMA effect estimate |  |  |
| Aiello 2023 | Melatonin / Ramelteon | Placebo | RR 0.76 (95% CI 0.54, 1.07); p=0.12; I^2^=64%; 6 studies (1625 participants); random effects model; very low certainty |  |  |
| **Other drugs** |  |  |  |  |  |
| Burry 2019 | Statin (HMG-CoA) | Placebo |  | RoM 1.05 (95% CrI 0.61, 1.77); direct evidence: 2 studies (414 participants); moderate certainty^2^  NMA effect estimate |  |
| **Sedation strategies** | | | | | |
| Herling 2018 | Protocolized sedation | Daily sedation interruption | RR 0.96 (95% CI 0.81, 1.14); p=0.65; I^2^=0%; 2 studies (483 participants); random effects model; very low certainty |  |  |
| Aitken 2021 | Light sedation | Deep sedation | RR 1.04 (0.88 to 1.23); p=0.65; I^2^=0%; 4 studies (556 participants); random effects model; moderate certainty |  |  |

| **Key:**  OR (Odds Ratio); CrI (Credible Interval); CI (Confidence Interval); NMA (Network Meta-Analysis); RR (Risk Ratio); MD (Mean Difference); d (days); RoM (Ratio of Means); SMD (Standardized Mean Difference); MRA (Melatonin Receptor Agonist); Green: Benefit (in favour of intervention); Grey: Favours neither the intervention or comparator; Blank cell: Outcome not assessed; * Cardiac surgical ICU |
| --- |
| **Footnotes:**  ^1^ One study with zero events in both arms excluded from effect estimate but not from total participants (?32 synthesised studies)  ^2^ Meta-analysis I^2^=0%  ^3^ Meta-analysis I^2^=82%  ^4^ Three different scales used (delirium rating scale; Memorial Delirium Assessment Scale; 4-point mental scoring scale).  ^5^ Forest plot result reported. Note difference in reporting of CI in-text although consistent direction of effect  ^6^ Severity defined as length of delirium episodes (no units) |
| **GRADE Working Group grades of evidence: High certainty / Moderate certainty / Low certainty / Very low certainty** |
