# Supplementary material for: A systematic meta-review of interventions to prevent and manage delirium in the Intensive Care Unit: Part 1 – Pharmacological interventions
Source: Crit Care. 2025 Dec 30;29:540. doi: 10.1186/s13054-025-05615-0 (PMC12751364; doi:10.1186/s13054-025-05615-0)
Supplement: Supplementary file 7 — Additional file 5: Pharmacological effect estimates for mapped ICU and hospital mortality outcomes. [file 13054_2025_5615_MOESM7_ESM.docx]

**Additional file 5: Pharmacological effect estimates for mapped ICU and hospital mortality outcomes**

| **Review** | **Drug / Drug class / Sedation strategy** | **Comparator** | **ICU mortality** (days or no units) | **Hospital mortality** (days or no units) |
| --- | --- | --- | --- | --- |
| **Alpha-2 adrenoceptor agonists / Dexmedetomidine** | | | | |
| Wang 2021 | Dexmedetomidine | Non-dexmedetomidine | RR 1.01 (95% CI 0.89, 1.14); p=0.90; I^2^=0%; 7 studies (4292 participants); random effects model; low certainty | RR 1.01 (95% CI 0.91, 1.12); p=0.88; I^2^=1%; 9 studies (5829 participants); random effects model; very low certainty |
| **Antipsychotics** | | | | |
| Herling 2018 | Haloperidol | Placebo |  | RR 0.98 (95% CI 0.80, 1.22); p=0.88; I^2^=0%; 2 studies (1580 participants); random effects model; moderate certainty^1^ |
| **Melatonergics** | | | | |
| Mukundarajan 2023 | Melatonin / Ramelteon | Placebo / Standard | OR 0.78 (95% CI 0.56, 1.11); p=0.17; I^2^=0%; 8 studies (1549 participants); fixed effects model; high certainty |  |
| **Sedation strategies** | | | | |
| Aitken 2021 | Light sedation | Deep sedation | RR 0.82 (95% CI 0.58, 1.17); p=0.28; I^2^=30%; 4 studies (725 participants); random effects model; moderate certainty | RR 0.93 (95% CI 0.75, 1.15); p=0.5; I^2^=0%; 5 studies (762 participants); random effects model; moderate certainty |
| Burry 2014 | Daily sedation interruption | No daily sedation interruption | RR 0.96 (95% CI 0.77, 1.21); p=0.75; I^2^=0%; 7 studies (815 participants); random effects model; moderate certainty |  |
| **Other** |  |  |  |  |
| Fraser 2013 | Non-benzodiazepine (dexmedetomidine or propofol) | Benzodiazepine (lorazepam or midazolam) |  | RR 1.01 (95% CI 0.78, 1.30); p=0.96; I^2^=31%; 5 studies (1101 participants); random effects model; moderate certainty^2,3^ |

|  |
| --- |
| \| **Key:**  RR (Risk Ratio); CI (Confidence Interval); OR (Odds Ratio); Grey: Favours neither the intervention or comparator; Blank cell: Outcome not assessed \| \| --- \| \| **Footnotes:**  ^1^ In-hospital mortality within 28 days  ^2^ In-hospital or up to 45 days after randomisation (‘short-term mortality’)  ^3^ Forest plot result reported. Note difference in reporting in-text although consistent direction of effect) \| \| **GRADE Working Group grades of evidence: High certainty / Moderate certainty / Low certainty / Very low certainty** \| |
