# Supplementary material for: A systematic meta-review of interventions to prevent and manage delirium in the Intensive Care Unit: Part 1 – Pharmacological interventions
Source: Crit Care. 2025 Dec 30;29:540. doi: 10.1186/s13054-025-05615-0 (PMC12751364; doi:10.1186/s13054-025-05615-0)
Supplement: Supplementary file 8 — Additional file 6: Pharmacological effect estimates for mapped ICU and hospital length of stay outcomes. [file 13054_2025_5615_MOESM8_ESM.docx]

**Additional file 6: Pharmacological effect estimates for mapped ICU and hospital length of stay outcomes**

| **Review** | **Drug / Drug class / Sedation strategy** | **Comparator** | **ICU LOS** (hours, days or no units) | **Hospital LOS** (hours, days or no units) |
| --- | --- | --- | --- | --- |
| **Alpha-2 adrenoceptor agonists / Dexmedetomidine** | | | | |
| Burry 2021 | ‘Alpha-2 adrenoceptor agonists’ | Placebo | RoM 0.78 (95% CrI 0.64, 0.95); 5 studies (1091 participants); moderate certainty  NMA estimate | RoM 0.65 (95% CrI 0.52, 0.83); 4 studies (1061 participants); moderate certainty  NMA estimate |
| Heybati 2022^*^ | Dexmedetomidine | Propofol | MD -8.94 h (95% CI: -22.40, 4.52); p=0.1603; I^2^=72%; 8 studies (466 participants); random effects model; very low certainty |  |
| Chen 2015 | Dexmedetomidine | Traditional (benzodiazepine or propofol or standard care (propofol and midazolam)) | Geometric MD -0.15 log d (95% CI -0.28, -0.01); p=0.04; I^2^=0%; 5 studies (1223 participants); random effects model; very low certainty |  |
| Fraser 2013 | Non-benzodiazepine (dexmedetomidine or propofol) | Benzodiazepine (lorazepam or midazolam) | MD -1.64 d (95% CI -2.57, -0.70); p=0.0006; I^2^=0%; 6 studies (1225 participants); random effects model; moderate certainty^1^ |  |
| Lewis 2021 | Dexmedetomidine | Mixed (other sedation strategies or placebo) | MD -2.40 d (95% CI -3.51, -1.29); p<0.0001; I^2^=83%; 6 studies (375 participants); random effects model; low certainty |  |
| Lewis 2022 | Dexmedetomidine | Mixed (other sedatives) | MD -0.32 d (95% CI -0.42, -0.22); p<0.00001; I^2^=85%; 47 studies (9472 participants); random effects model; low certainty | MD 0.10 d (95% CI -0.72, 0.91); p=0.82; I^2^=66%; 21 studies (7279 participants); random effects model; moderate certainty |
| Zhang 2022 | Dexmedetomidine | Mixed (other sedatives) | SMD -0.22 (95% CI -0.85, 0.41); p=0.5; I^2^=0%; 9 studies (659 participants); fixed effects model; high certainty^2^ |  |
| Wang 2021 | Dexmedetomidine | Mixed (non-dexmedetomidine) | MD -0.91 d (95% CI -1.28, -0.54); p<0.00001; I^2^=94%; 26 studies (7876 participants); random effects model; very low certainty | MD -1.98 d (95% CI -3.41, -0.55); p=0.007; I^2^=95%; 19 studies (7736 participants); random effects model; very low certainty |
| **Antipsychotics** | | | | |
| Burry 2021 | All | Placebo | RoM 1.01 (95% CrI 0.87, 1.18); 8 studies (2776 participants); low certainty  NMA estimate | RoM 1.01 (0.85, 1.21); 6 studies (2668 participants); low certainty  NMA estimate |
| Burry 2019 | Typical | Placebo | RoM 0.99 (95% CrI 0.85, 1.17); 4 studies (618 participants); moderate certainty^3^  NMA estimate | RoM 0.92 (95% CrI 0.65, 1.18); 2 studies (479 participants); low certainty^4^  NMA estimate |
| Burry 2019 | Atypical | Placebo | RoM 0.92 (95% CrI 0.80, 1.08); 4 studies (577 participants); high certainty^3^  NMA estimate | RoM 0.93 (95% CrI 0.69, 1.16); 3 studies (511 participants); moderate certainty^3^  NMA estimate |
| Herling 2018 | Haloperidol | Placebo | MD 0.18 d (95% CI -0.6, 0.97); p=0.64; I^2^=8.65%; 2 studies (1580 participants); random effects model; high certainty^2^ |  |
| **Melatonergics** | | | | |
| Mukundarajan 2023 | Melatonin / Ramelteon | Placebo / Standard | MD 0.05 no units (95% CI -0.65, 0.75); p=0.89; I^2^=36%; 10 studies (1638 participants); random effects model; high certainty | MD -1.46 no units (95% CI -4.50, 1.59); p=0.37; I^2^=0%; 2 studies (144 participants); fixed effects model; moderate certainty^2^ |
| Burry 2021 | Melatonin / MRA | Placebo | RoM 0.95 (95% CrI 0.75, 1.20); 4 studies (488 participants); low certainty  NMA estimate | RoM 0.92 (0.64, 1.32); 2 studies (340 participants); moderate certainty  NMA estimate |
| Aiello 2023 | Melatonin / Ramelteon | Placebo | MD -0.26 d (95% CI -0.89, 0.37); p=0.42; I^2^=75%; 8 studies (1453 participants); random effects model; very low certainty |  |
| **Opioids** | | | | |
| Yang 2021 | Remifentanil | Other opioids | SMD -0.33 (95% CI -0.60, -0.07); p=0.01; I^2^=50%; 13 studies (?1055 participants); random effects model; low certainty^5^ | SMD -0.29 (95% CI -0.67, 0.10) p=0.15; I^2^=74%; 4 studies (?432 participants); random effects model; very low certainty^6^ |
| **Other drugs** |  |  |  |  |
| Burry 2019 | Statin | Placebo |  | RoM 0.98 (95% CrI 0.69, 1.30); 2 studies (369 participants); moderate certainty^3^  NMA estimate |
| **Sedation strategies** | | | | |
| Aitken 2021 | Light sedation | Deep sedation | MD 0.28 d (95% CI -1.46, 2.02); p=0.75; I^2^=32%; 6 studies (1462 participants); random effects model; moderate certainty | MD -0.69 d (95% CI -6.96, 5.58); p=0.83: I^2^=80%; 5 studies (762 participants); random effects model; very low certainty |
| Burry 2014 | Daily sedation interruption | No daily sedation interruption (usual care or other protocolised sedation strategies) | Geometric MD -6% (95% CI -19, 8); MD -0.1 log d (95% CI -0.22, 0.03); p=0.13; I^2^=45.2%; 9 studies (1282 participants); random effects model; moderate certainty | Geometric MD -2% (95% CI -16, 14); MD -0.06 log d (95% CI -0.20, 0.08); p=0.44; I^2^=39.16%; 8 studies (1232 participants); random effects model; moderate certainty |
| Herling 2018 | Daily sedation interruption | Protocolised sedation | MD -1.19 (95% CI -2.91, 0.53); p=0.18; I^2^=0%; 2 studies (483 participants); random effects model; very low certainty |  |

|  |  |
| --- | --- |
|  |  |
| **Key:**  LOS: length of stay; RoM (Ratio of Means); CrI (Credible Interval); NMA (Network Meta-Analysis); MD (Mean Difference); h (hours); CI (Confidence Interval); d (days); SMD (Standardized Mean Difference); MRA (Melatonin Receptor Agonist); Green: Benefit (in favour of intervention); Grey: Favours neither the intervention or comparator; Blank cell: Outcome not assessed; * Cardiac surgical ICU | |
| **Footnotes:**  ^1^ Forest plot result reported. Note difference in reporting in-text difference although consistent direction of effect  ^2^ Forest plot result reported. Note difference in reporting in-text with inconsistent direction of effect  ^3^ Pairwise meta-analyses I^2^=0%  ^4^ Pairwise meta-analysis I^2^=72%  ^5^ Forest plot result included 15 comparisons from 13 studies (Engoren 2001 a&b; Karabinis 2004 a&b) and suggests a unit of analysis issue with multi-arm studies (double-counting) affecting the overall effect estimate; in-text reporting of 1034 participants  ^6^ Forest plot result included 5 comparisons from 4 studies (Engoren 2001 a&b) and suggests a unit of analysis issue with a multi-arm study (double-counting) affecting the overall effect estimate; in-text reporting of 264 participants | |
| **GRADE Working Group grades of evidence: High certainty / Moderate certainty / Low certainty / Very low certainty** | |
