# Supplementary material for: A systematic meta-review of interventions to prevent and manage delirium in the Intensive Care Unit: Part 1 – Pharmacological interventions
Source: Crit Care. 2025 Dec 30;29:540. doi: 10.1186/s13054-025-05615-0 (PMC12751364; doi:10.1186/s13054-025-05615-0)
Supplement: Supplementary file 9 — Additional file 7: Types of adverse events reported in pharmacological reviews of ICU delirium. [file 13054_2025_5615_MOESM9_ESM.docx]

**Additional file 7: Types of adverse events reported in pharmacological reviews of ICU delirium**

***** Occasionally delirium reported as part of adverse events. ** Delirium and coma-free days also reported as a composite outcome in some evidence reviews.
